# Unique molecular identifiers don’t need to be unique: a collision-aware estimator for RNA-seq quantification

**DOI:** 10.1101/2025.09.08.674884

**Published:** 2026-05-20

**Authors:** Dylan Agyemang, Rafael A. Irizarry, Tavor Z. Baharav

**Affiliations:** 1 Department of Statistics, University of North Carolina at Chapel Hill, Chapel Hill, NC, USA.; 2 Department of Biostatistics, Harvard T.H. Chan School of Public Health, Boston, MA, USA.; 3 Department of Data Science, Dana-Farber Cancer Institute, Boston, MA, USA.; 4 Eric and Wendy Schmidt Center, Broad Institute, Cambridge, MA, USA.

**Keywords:** Single-cell RNA sequencing, Unique Molecular Identifier, method-of-moments

## Abstract

RNA-sequencing (RNA-seq) relies on Unique Molecular Identifiers (UMIs) to accurately quantify gene expression after PCR amplification. Longer UMIs minimize collisions—where two distinct transcripts are assigned the same UMI—at the expense of increased sequencing and synthesis costs. However, it is not clear how long UMIs need to be in practice, especially given the nonuniformity of the empirical UMI distribution. In this work, we develop a method-of-moments estimator that accounts for UMI collisions, accurately quantifying gene expression and preserving downstream biological insights. We show that UMIs need not be unique: shorter UMIs can be used with a more sophisticated estimator.

## 1 Introduction

**Fig. 1:**
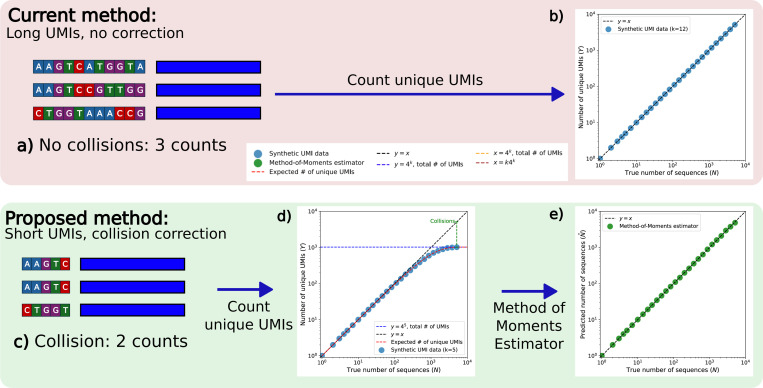
Comparison between the standard pipeline and our collision-aware estimator. Synthetic data, details in Section S1.6. Collisions occur when distinct transcripts are assigned identical UMI sequences. **a)** With long UMIs (k=12) collisions are rare. All 3 transcripts (dark blue rectangles) are assigned different UMIs, and so deduplication yields 3 counts. **b)** Deduplicated UMI counts accurately predict the true number of transcripts for long UMIs. **c)** With short UMIs (k=5) collisions will occur. Here, 3 transcripts are sequenced, but 2 are assigned the same UMI, leading to a deduplicated count of 2. **d)** Deduplicated UMI counts fall far below the y=x line due to collisions. Our statistical model (red line) accurately predicts this observed relationship. **e)** Our method-of-moments estimator applied to **d** corrects for these collisions.

RNA-sequencing is widely used to study the sequence and abundance of mRNA molecules within cells [1, 2]. In order to generate sufficient genomic material for sequencing, especially at single-cell resolution [3], mRNA molecules must be PCR amplified. However, the nonuniform amplification of PCR makes UMIs essential for accurate quantification [4]. UMIs are randomly generated nucleotide sequences of a specific length that are attached to the reverse-transcribed cDNA molecules in the sequencing process [5]. After PCR amplification, reads that originate from the same molecule will carry the same UMI (Figure 1a). Genomic analysis pipelines [3] identify reads with the same sequence and same UMI as duplicates, and collapse them into a single count. Reads with distinct UMIs but identical sequences are counted separately (Figure 1b). However, the reliability of UMIs to correctly make this distinction depends on the length of the UMI used. At each position of a UMI, we have a choice of 4 nucleotides, yielding a total of $K=4^{k}$ distinct length-k UMIs. With shorter UMI lengths we observe *collisions*, where multiple identical mRNA transcripts are assigned the same UMI sequence [6] (Figure 1c). Consequently, deduplication leads to an underestimation of the true transcript abundance, as it misattributes the collision to a PCR duplicate rather than a distinct additional transcript (Figure 1d). With longer UMIs we avoid these collisions, at the expense of additional sequencing cycles and increased synthesis difficulty. As it stands, there is no consensus on how to choose UMI length to balance cost and quantification accuracy, so varied lengths are used in practice. For example, the UMI length was notably increased from 10-bp in 10x Chromium v2 to 12-bp in 10x Chromium v3, while 8-bp was originally used in scRNA-seq [7] and 6-bp or 8-bp is often still used in miRNA experiments [8].

Several prior works have studied the statistical and computational challenges arising from UMIs. The classical occupancy framework underlying UMI collisions was introduced by Fu et al. [6], who derived the expected number of unique labels (UMIs) observed as a function of the number of molecules and the label pool size, assuming a uniform distribution over labels. Kivioja et al. [5] formalized UMIs as a general tool for absolute molecular quantification in genome-scale sequencing, demonstrating their utility in digital karyotyping and mRNA-seq. Clement et al. [9] formalized collision probabilities and allelic fraction distortion for amplicon sequencing, providing guidelines for choosing UMI lengths that avoid collisions entirely, but did not develop an estimator that corrects for collisions after the fact. Substantial effort has been devoted to the upstream problem of UMI error correction: graph-based approaches [10], alignment-free methods [11], Phred quality-based methods [12], and Hamming distance-based deduplication [5, 7]. However, error correction methods are not designed to account for collisions, and can even exacerbate underestimation with short UMI lengths by over-collapsing UMIs that are close in Hamming distance but actually represent distinct transcripts (Section S1.3). The most comprehensive prior treatment is dropEst [13], which jointly models UMI error correction, deduplication, and collision adjustment under a nonuniform UMI distribution. However, the dropEst model is computationally intensive, requiring quantization and approximate computation. It does not admit a rigorous theoretical analysis of estimator performance or estimation limits, and because it operates as an end-to-end pipeline from raw reads to final count matrix, it cannot be applied as a modular plug-in to standard pipelines or benchmarked on synthetically shortened UMIs. Recent work has also identified that the empirical nonuniformity of UMI distributions arises in part from truncated oligonucleotide synthesis on beads [14], a mechanistic insight we incorporate into our statistical model (Section 4.1).

In this work, we address these gaps and develop a method-of-moments estimator for the true transcript abundance that accounts for UMI collisions, is near-optimal, is computationally efficient, and is designed as a modular plug-in for existing pipelines. Expanding on the basic UMI collision model [6], we derive a simple closed-form estimator in the uniform case and an efficiently computable estimator in the nonuniform case, requiring only a one-time lookup table precomputation. Leveraging classical statistical results [15], we show that our estimator essentially matches the Cramér–Rao lower bound in a simplified binomial setting (Theorem 1). In the Poissonized setting, we derive the maximum likelihood estimator (MLE), which uses information about *which* UMIs were observed, not just the number of unique ones, and show that our method-of-moments estimator is optimal when p is uniform, with a characterizable efficiency gap that grows with the skewness of p (Section S3). However, in practice the method-of-moments estimator performs essentially as well as the MLE, in a much more computationally efficient manner (Sections S3 and S4). We characterize the threshold for nontrivial estimation, showing that beyond the coupon collector [16] threshold of KlogK transcripts, accurate estimation is impossible (Proposition 2). We provide asymptotic confidence intervals via the delta method (Section S5.2). Going beyond the uniform assumption of prior works, we develop a mechanistic statistical model of UMI nonuniformity that accounts for failures in UMI synthesis (Section 4.1). Our estimator is computationally efficient (precompute lookup table, then constant time evaluation) and works in concert with existing UMI error correction methods, where error correction serves as a preprocessing step and our collision adjustment is applied afterward. This modularity, absent from prior collision-aware approaches, enables straightforward integration into any existing RNA-seq pipeline and allows us to validate performance on synthetically shortened UMIs. Our estimator allows researchers to reclaim 6 base pairs from the UMI for biological payload or additional barcodes, without losing any quantification accuracy. This can be critical in spatial barcoding and combinatorial indexing, which require extensive sequencing real estate.

## 2 Problem formulation

Methodologically, in this work we study, for a given gene, how to estimate the number of unique pre-amplification mRNA molecules. Specifying to one particular mRNA molecule, we make the assumption that the UMI sequence does not impact its amplification. This assumption is reasonable, as the UMI is a short sequence (up to 12 bp) attached to a much longer cDNA transcript (several hundred base pairs), and is therefore unlikely to impact amplification. This motivates our statistical model where each mRNA molecule is tagged with a UMI randomly drawn from some distribution over the $4^{k}$ possible UMIs, and that each pre-amplification mRNA molecule has an equal probability of appearing in the set of sequenced reads.

Under these assumptions, we model our problem on a gene-by-gene basis using the classical balls and bins framework. Consider N identical balls randomly assigned into K bins, with the observation Y denoting the number of bins with at least one ball. In our sequencing model, this translates to N mRNA transcripts for a given gene before PCR amplification, $K=4^{k}$ possible UMIs, and Y observed unique UMIs. Given the UMI distribution p over the K UMIs, we want to estimate the number of transcripts N after observing Y=y unique UMIs. This estimation question relates to the classical occupancy problem [15]. Naively, one would estimate $\hat{N}(y)=y$, which is suboptimal as for $N>\sqrt{K}$ (the birthday paradox [16]) collisions occur with probability greater than 1/2. More importantly, estimation is possible up to the coupon collector threshold of N=KlogK, the expected number of original mRNA transcripts that need to be sequenced in order for us to observe all K UMIs [16]. We show that N cannot be consistently estimated above this threshold, and conversely that below this threshold our estimator provides excellent performance, theoretically matching the Cramér–Rao lower bound in a simplified binomial setting [17] (Section S7). These results simplify nicely when all UMIs are equally likely, but in practice UMIs do not follow a uniform distribution, which biases the estimator if unaccounted for. We adapt our estimator to this nonuniform case, yielding an efficiently computable estimator $\hat{N}(\cdot )$ which we derive below.

## 3 Abundance estimator

Recall our statistical model, restated here for completeness: N identical balls are randomly assigned into K bins, with the observation Y denoting the number of bins with at least one ball. In our sequencing model, this translates to N mRNA transcripts before PCR amplification, $K=4^{k}$ possible UMIs, and Y unique UMIs observed. The N balls (transcripts) which we denote by $X_{i}$, follow some distribution p over the set [K]={1,2,…,K}, and are independent and identically distributed. Then:

(1) $$X_{1},X_{2},\ldots ,X_{N}\overset{\text{i.i.d.}}{∼}p,$$

(2) $$Y=\text{\# unique}\left\{X_{1},X_{2},\ldots ,X_{N}\right\}.$$

Mathematically, $Y=\left|\left\{X_{1},X_{2},\ldots ,X_{N}\right\}\right|$, where |S| denotes the cardinality of a set S, in our case the number of distinct UMIs observed. Given p and Y, we want to estimate N. By representing Y as a sum of K indicators, we can compute its mean and variance.


(3) $$E[Y]=K-\sum_{j=1}^{K}{\left(1-p_{j}\right)}^{N}\approx K-\sum_{j=1}^{K}e^{-p_{j}N}$$

(4) $$\text{Var}(Y)=\sum_{j=1}^{K}\left[{\left(1-p_{j}\right)}^{N}-{\left(1-p_{j}\right)}^{2N}\right]+\sum_{j\neq k}\left[{\left(1-p_{j}-p_{k}\right)}^{N}-{\left(1-p_{j}\right)}^{N}{\left(1-p_{k}\right)}^{N}\right]\;\;\approx \sum_{j=1}^{K}\left[e^{-p_{j}N}-e^{-2p_{j}N}\right]+\sum_{j\neq k}\left[-Np_{j}p_{k}e^{-N\left(p_{j}+p_{k}\right)}\right]$$

A second order Taylor approximation is necessary to simplify the crossterms in the variance. Observe that E[Y] is a concave function of p for N≥1, as can be verified by taking the second derivative. By Jensen’s inequality, this implies that the expected number of unique UMIs observed is maximized when p is uniform.

This assumption that the $X_{i}$ are independent and identically distributed need not perfectly hold in practice. For example, sequencing errors may introduce dependencies between the $X_{i}$ (Section S1.3). Additionally, the UMI distribution may vary across genes, due to binding affinities; however, as we show in Figure 4a, all genes appear to follow the same UMI distribution, except for MALAT1. Overall, this simple model in Equations (1) and (2) fits the data quite well with a common UMI distribution p in practice, and we discuss estimating p and identifying the deviation of MALAT1 in Section 4.

### 3.1 Simplification in uniform setting

Simplifying Equation (3) in the case when all UMIs are equally likely we recover a classical result [6]. Rearranging the expectation of Y, we obtain our method-of-moments estimator in the uniform UMI setting, when we observe Y=y unique UMIs:

(5) $$E[Y]=K\left(1-{\left(1-\frac{1}{K}\right)}^{N}\right)\approx K\left(1-e^{-N/K}\right)$$

(6) $$\hat{N}(y)=\left\{\begin{array}{ll} \frac{\log(1-y/K)}{\log(1-1/K)} & \text{when}\;y<K,\\ \hat{N}(K-1)+K & \text{when}\;y=K. \end{array}\right.$$

This choice of $\hat{N}(K)=\hat{N}(K-1)+K$ is motivated by the fact that the expected number of additional transcripts required to go from observing K−1 unique UMIs to K unique UMIs is K. This extension of $\hat{N}$ retains the convexity of the estimator (Proposition 1). Extrapolation is an edge case that should be rarely used in practice: if Y=K is observed then k is likely too small for the given sequencing depth.

This balls and bins setting is known to have several interesting thresholds, considering the case of large K [16]. First is the classical birthday paradox, which occurs at $N=\sqrt{K}$. At this point, collisions occur with probability greater than 1/2, indicating that the naive estimator will underestimate the true counts over half the time. Next is the value of N=K. This is the maximum counts that the naive estimator will output, where we note that at this value of N, $E[Y]\approx \left(1-e^{-1}\right)N\approx .63N$, indicating the dramatic bias of the collision-oblivious (naive) estimator. Finally, we have the coupon collector threshold of N=KlogK, the expected number of unique mRNA transcripts that need to be sequenced in order for our observations to saturate with Y=K, where *all* K UMIs are observed. We show that this is the true threshold for consistent estimation, where for N>KlogK we have Y=K with high probability (Proposition 2). This implies that all K UMIs are observed, beyond which larger N cannot be estimated. Surprisingly, we show that for N≤cKlogK for c<1, our estimator performs near optimally, with mean squared error (MSE) matching the Cramér–Rao lower bound [17] in a simplified binomial setting (Theorem 1).

### 3.2 Method-of-moments estimator (nonuniform)

While our estimator in the previous subsection is clean, it does not account for the nonuniformity of UMI distributions in practice (discussed in more detail in Section 4). In the nonuniform case with probabilities ${\left\{p_{j}\right\}}_{j=1}^{K}$ the expected number of unique UMIs of length k observed, given that there are Computation and evaluation of our proposed collision-aware estimator. Algorithm 1 details the overall procedure, while Algorithm 2 describes the precomputation of the estimator $\hat{N}(\cdot )$. Note that after precomputation, each count can be mapped to its collision-corrected estimate in constant time. Algorithm 1 is applied after UMI error correction and deduplication, before downstream analysis (additional runtime and computational complexity analysis in Section S5.3).

**Algorithm 1:** Collision-aware estimator

1:	**Input:** Cell x gene counts matrix $X\in {\mathbb{N}}^{n\times d}$
2:	*Optional: compute dataset-specific UMI distribution* p. *By default use* Eq. (8).
3:	$Y_{\max}$ ← maximum observed count in X.
4:	Precompute $\hat{N}(\cdot )$ using Algorithm 2.
5:	$\tilde{X}$ ← apply $\hat{N}(\cdot )$ elementwise to X.
6:	**Output:** Collision-corrected counts matrix $\tilde{X}$

**Algorithm 2:** Precomputation of $\hat{N}(y)$

1:	**Input:** UMI distribution p, max count $Y_{\max}$
2:	n←1
3:	**repeat**
4:	Compute $Y_{n}=\sum_{j=1}^{K}\left(1-{\left(1-p_{j}\right)}^{n}\right)$
5:	n←n+1
6:	**until** $Y_{n-1}>\min\left(Y_{\max},K-1\right)$
7:	Initialize array $\hat{N}(0),\ldots ,\hat{N}\left(Y_{\max}\right)$ with zeros
8:	$n_{\text{ptr}}\leftarrow 1$ ▷ Pointer for $\left(Y_{n}\right)$
9:	**for** $y=1,\ldots ,\min\left(Y_{\max},K-1\right)$ **do**
10:	**while** $n_{\text{ptr}}+1<n$ **and** $Y_{n_{\text{ptr}}+1}\leq y$ **do**
11:	$n_{\text{ptr}}\leftarrow n_{\text{ptr}}+1$
12:	**end while** ▷ Now $Y_{n_{\text{ptr}}}\leq y<Y_{n_{\text{ptr}}+1}$
13:	$\hat{N}(y)\leftarrow n_{\text{ptr}}+\frac{y-Y_{n_{\text{ptr}}}}{Y_{n_{\text{ptr}}+1}-Y_{n_{\text{ptr}}}}$ ▷ Eq. (7)
14:	**end for**
15:	If $Y_{\max}=K$, set $\hat{N}(K)$ using Eq. (7)
16:	**Output:** Array $\hat{N}(0),\ldots ,\hat{N}\left(Y_{\max}\right)$

N transcripts before PCR amplification, is $Y_{N}=E[Y]=K-\sum_{j=1}^{K}{\left(1-p_{j}\right)}^{N}$ (from Equation (3)). Inverting this expression to solve for N as a function of Y does not yield a closed form solution. However, given that $Y_{N}$ is monotonically increasing in N (and concave), we can define $\hat{N}(y)$ by identifying n such that $Y_{n}\leq y\leq Y_{n+1}$, and linearly interpolating. Since $p_{j}>0$ for all j, $Y_{N}$ is concave, strictly increasing in N, and twice differentiable. Thus, $\hat{N}(y)$ is convex in y for y<K, as it is the linear interpolation between sampled points of a convex function. As before, the case of y=K is a priori undefined, as $Y_{n}<K$ for all n. Here we use a quadratic extrapolation based on finite differences to yield a simple estimate that retains the convexity of our estimator (details in Section S5.1.1). As before, this extrapolation is an edge case that should be rarely used in practice. Concretely, our final estimator is given by:

(7) $$\hat{N}(y)=\left\{\begin{array}{ll} n+\frac{y-Y_{n}}{Y_{n+1}-Y_{n}},\;\text{where}\;n\;\text{satisfies}\;Y_{n}\leq y<Y_{n+1} & 0\leq y<K,\\ 3\hat{N}(K-1)-3\hat{N}(K-2)+\hat{N}(K-3), & y=K. \end{array}\right.$$

We can approximate the variance of our estimator $\hat{N}(y)$ using the delta method. Leveraging the fact that our estimator is (up to linear interpolation) the inverse function of $Y_{N}$, we obtain that $\text{Var}(\hat{N}(Y))\approx {\left({\hat{N}}^{\prime }(Y)\right)}^{2}\text{Var}(Y)$. This enables us to compute confidence intervals for our predictions $\hat{N}(y)$ (details in Section S5.2). From classical literature, we know that Y is asymptotically normally distributed, as long as Var(Y)→∞ [15]. This holds for $N=\omega (\sqrt{K})$, once collisions become non-negligible, up until N=o(KlogK), before saturation occurs at the coupon collector threshold (details in Section S7). This enables us to quantify the bias of our estimator, which is vanishing for N=o(K) (see Table 1 for full N results).

Our estimator can be implemented very efficiently as a modular plug-in to existing RNA-seq pipelines. Given a cell x gene counts matrix $X\in {\mathbb{N}}^{n\times d}$ of per-gene error-corrected and deduplicated UMI counts, we can compute with Algorithm 1 a collision-corrected counts matrix $\tilde{X}\in {\mathbb{R}}^{n\times d}$. This is done by precomputing the estimator $\hat{N}(\cdot )$ for all observed counts up to the maximum observed count $Y_{\max}$ in X using Algorithm 2, and then applying $\hat{N}(\cdot )$ elementwise to X. This precomputation step only needs to be done once per dataset, and each count can then be mapped to its collision-corrected estimate in constant time.

## 4 Nonuniformity of empirical UMI distribution

**Fig. 2:**
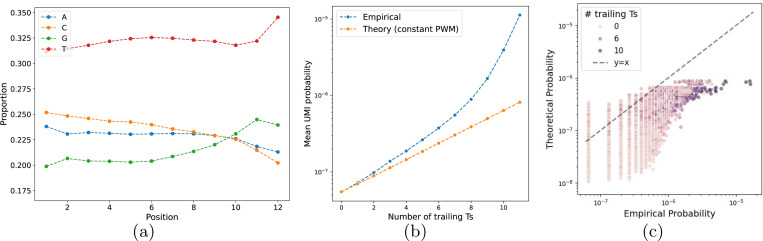
Analysis of empirical UMI nucleotide frequencies in 1k PBMC dataset. **a)** Position weight matrix (PWM) for the empirical UMI distribution across all genes except MALAT1. **b)** Mean probability for all UMIs given a certain number of trailing Ts. This is shown for the empirical frequencies of non-MALAT1 genes (blue), as well as the theoretical probabilities based on the constant PWM (Equation (8), orange). MALAT1 and PWM from **a)** are shown in Figure S6. **c)** Empirical UMI probabilities versus predicted probabilities using the constant PWM model in Equation (8).

As identified by previous works, UMIs in practice follow a nonuniform distribution [9, 13, 14], which holds consistently across different runs of the same chemistry and so can be estimated from data. This nonuniformity holds across chemistry choices, where drop-Seq UMIs exhibit dramatically higher G frequencies [14]. In this work, we focus primarily on 10X Genomics kits, where this distribution is T-biased (elevated T frequency), and is relatively well approximated by a marginal per-base model, where the probability of the UMI depends only on its aggregate nucleotide composition (Figure S1). The only exception is UMIs with many trailing Ts, which are significantly more likely (Figure 2b,c). Recent work has shown that this is due to *truncated UMI synthesis* [14]; while all UMIs are supposed to be the same length (e.g. k=12 bp), some miss several rounds of synthesis and end up as length 8. Then, due to the structure of 10x’s beads, the sequencer will keep reading into the 30-bp poly(dT) tail [18], mistakenly reading 4 Ts as the last 4 bases of the UMI. All genes follow the same trend, except for MALAT1, which we are able to identify and filter out (Section 4.2). Examining our 1k PBMC dataset, we compute the following position weight matrix characterizing the marginal probability distribution at each base pair of the UMI (Figure 2a).

The UMI frequencies can be well modeled by an independent, per-base probability distribution, which improves for shorter UMI lengths (Figure S1). For generating synthetic data with shortened UMI lengths, we truncate and only look at the first k base pairs of the UMI, as this mimics the true UMI synthesis process (discussed in Section S1.2). We model this probability by defining $n_{L}$ as the number of occurrences of nucleotide L∈{A,C,G,T} in the UMI sequence S. Probabilistically, this independent model states that:

(8) $$\mathbb{P}(S)=p_{A}^{n_{A}}p_{C}^{n_{C}}p_{G}^{n_{G}}p_{T}^{n_{T}},\;\text{where}\left[p_{A},p_{C},p_{G},p_{T}\right]=[.23,.24,.21,.32].$$

The per nucleotide probabilities are fit from the 1k PBMC dataset (Figure 2a), and hold stable across other 10x datasets with the same chemistry [14]. For non-10x chemistries, the specific probabilities may differ, but the same modeling approach (computing the empirical nucleotide frequencies and using Equation (8)) can be applied.

### 4.1 Failure in UMI synthesis

The only UMIs that significantly deviate from the model in Equation (8) are those that end with a string of Ts, as shown in Figure 2b,c. This is due to truncation during UMI synthesis, and with the 10x chemistry of 3′ sequencing, this leads us to read into the poly(dT) tail [14]. This is because of the specifics of 10x’s beads: to capture the mRNA transcript, each bead contains (in order) a TruSeq Read 1, the cell barcode (16-bp), the UMI (supposed to be 12-bp), poly(dT) sequence (30-bp), and finally VN (1-bp) an anchor to indicate the end of the poly(dT) stretch. However, in the sequential synthesis of UMIs, not all will make it to the full length 12. In fact, the authors claim that under 50% of 10x Chromium beads have the claimed length of 28 (16-bp barcode plus 12-bp UMI).

Mechanistically, at each iteration of UMI synthesis, there is some probability of failure to add the next base pair. In this case the UMI is capped at the end of that iteration, and no further base pairs are added. Assuming that the failure probability q is constant across all base pairs, this leads to a geometric distribution on the length of the synthesized UMI. When the UMI is shorter than 12-bp, the sequencer will read into the poly(dT) tail, leading to extra Ts at the end of the observed UMI. We show this process schematically in Figure S3, and refer the reader to [14] for further biochemistry details. Thus, we obtain the explicit probability of observing a UMI S with T trailing Ts by summing over all possible synthesis lengths ℓ from 12 − T to k.


(9) $$\mathbb{P}(S)=\sum_{\ell =k-\text{T}}^{k}\mathbb{P}\left(S_{:\ell }\right){\left(1-q\right)}^{\ell }q^{𝟙\{\ell <k\}}=\sum_{\ell =k-\text{T}}^{k}\prod_{i=1}^{\ell }\mathbb{P}\left(S_{i}\right){\left(1-q\right)}^{\ell }q^{𝟙\{\ell <k\}}$$

**Fig. 3:**
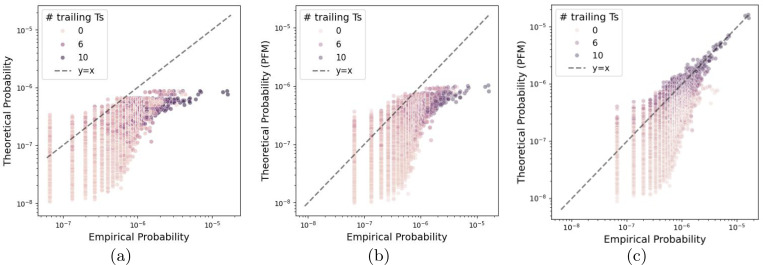
Fit of empirical 12-bp UMI probabilities using different models. **a)** Prediction using a constant PWM as in Equation (8). **b)** Prediction using PWM in Figure 2a. **c)** Prediction using truncated UMI synthesis model in Equation (9), monotonic per-bp synthesis probabilities fit as detailed in Section S1.2.

This model can be extended to allow for position-dependent failure probabilities $q_{i}$, which when fitted to the data yield an extremely good fit (Figure 3c), details in Section S1.2. As these UMIs with many trailing Ts constitute a small fraction of all UMIs, we retain the simpler model in Equation (8) for computational and conceptual simplicity.

### 4.2 Poor fit of MALAT1

**Fig. 4:**
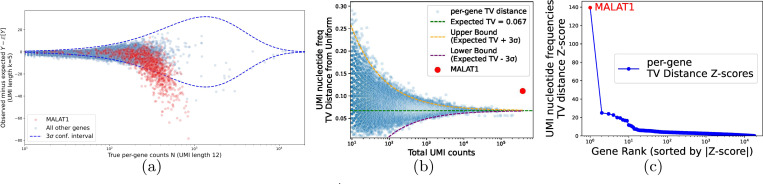
Outlier detection of MALAT1. **a)** 10× 1k PBMC dataset, corrected and deduplicated UMI counts. Nonuniform model (Equation (3)) with UMI frequencies generated by the marginal per-base pair model (Equation (8)). 3σ confidence intervals constructed from Equation (4) using nonuniform model. All genes aside from MALAT1 fall well within the 3σ confidence intervals. **b)** Per-gene analysis of TV distance between UMI nucleotide frequencies and mean nucleotide frequencies. Observed data is slightly overdispersed relative to the multinomial model, but MALAT1 is a clear outlier. **c)** Z-score computed for each gene’s TV distance, with variance approximation from Equation (12) (details in Section S1.5). Sorted z-scores validate the observation from **b)** that MALAT1 is a statistical outlier.

Analyzing the observed counts for shortened UMIs, our nonuniform model displays good accuracy across genes. However, we identify in this work that one gene, MALAT1, is a consistent outlier. Formally, studying the 1k PBMC dataset (Figure 4a), we observe that almost all counts fall within 3σ confidence intervals of their expectation under our model, except for MALAT1 (colored in red). As can be seen, these points follow a fundamentally different trend, and are negatively biased (i.e. experience more collisions). This same behavior was identified across all datasets studied.

MALAT1, a long non-coding RNA, is notorious in the literature for its high expression, high rate of internal priming, and lack of polyadenylation [19, 20]. MALAT1 expression is commonly used for quality control [19], as it is retained within the cell nucleus, and so if high MALAT1 expression is not detected within a droplet, then the droplet is likely either empty or contains a poor-quality cell. MALAT1 does not have a poly(A) tail, but rather folds onto itself to form a unique and highly stable triple-helical A-rich structure [20].

We show that this deviation of MALAT1 can be detected de novo, directly from its UMI distribution (Figure 4). Concretely, in these plots we compute for each gene its empirical UMI distribution across all cells, and collapse this to a per-nucleotide distribution summed across all 12 positions. This yields $12\tilde{Y}$ counts for $\tilde{Y}$ 12-bp UMIs observed across all cells for this gene. After normalization, we compute the total variation (TV) distance between each gene’s per-nucleotide UMI distribution and the expected distribution (summing counts across all cells and all genes). Plotting this for all genes reveals MALAT1 as a clear outlier (Figure 4a). To provide confidence bands on the TV distance in Figure 4b, we formulate a simple null model that UMIs are i.i.d. from a common per-nucleotide distribution (details in Section S1.5). While reality is overdispersed relative to this simple null, it provides a useful framework for identifying outliers. Computing the z-score of each gene’s TV distance immediately identifies MALAT1 as the only gene that deviates significantly from the expected distribution (Figure 4c). Because of this, we exclude MALAT1 from further analyses: if desired, our method can be applied to MALAT1 using a separate MALAT1-specific UMI distribution. Because our model explicitly accounts for sequence composition, it can automatically flag biological anomalies like internal priming (MALAT1), acting as a quality control metric that naive counting may miss.

## 5 Estimator Validation

**Fig. 5:**
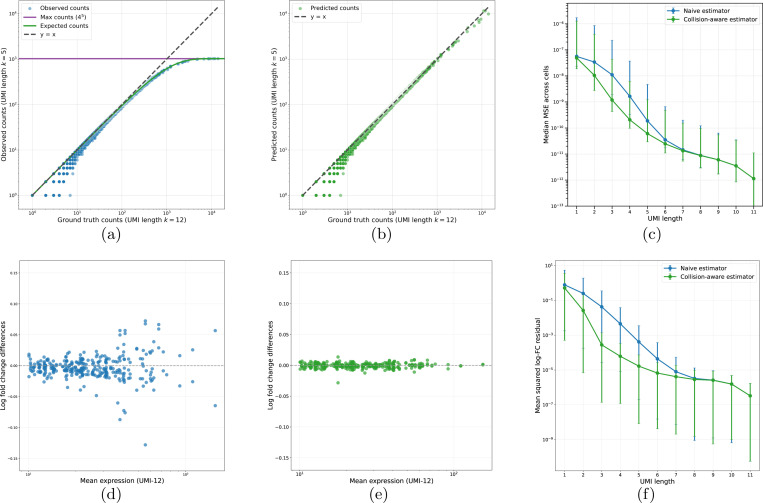
Improved performance of method-of-moments estimator on 10x’s PBMC 1k dataset. Panels **a-c** demonstrate enhanced accuracy in raw expression estimation, while panels **d-f** show the downstream improvement for log-fold change (LFC) estimation of differentially expressed genes (all genes shown in Figure S11). Processing details in Section S1. **a)** Concordance between our statistical model of collisions and the observed data for a UMI length of k=5. **b)** Same as **a** but using our collision-aware estimator. MSE is reduced by 95% from **a** to **b**. **c)** Per-cell MSE between normalized gene expression vectors estimated from shortened UMIs and the ground truth (k=12), shown as a function of UMI length (median and 95% CIs). **d)** Difference between predicted (using k=5, naive estimator) and true LFC (predicted - ground truth). **e)** Same as **d** but using our collision-aware estimator. MSE is reduced by 96% from **d** to **e**. **f)** MSE for estimating LFC (as in **d,e**), evaluated across UMI lengths (95% CIs).

To validate the performance improvement afforded by our method, we empirically evaluated our collision-aware estimator on three publicly available 10x Genomics Peripheral Blood Mononuclear Cells (PBMC) datasets, of 1k (Figure 5) and 10k (Figure S7) cells sequenced using Chromium Single Cell 3′ v3 chemistry, and of 5k (Figure S8) cells sequenced using v4 chemistry. For each dataset, we artificially shortened the error-corrected length 12 UMIs to length k=1,…,12. For each UMI length, we utilized our estimator to predict the true collision-free counts from the number of unique length k UMIs observed (details in Section S1). As the ground truth, we used the counts obtained from the full length 12 UMIs, which are largely collision-free; for a uniform UMI distribution, the expected number of unique length 12 UMIs (from Equation (5)) observed for N=1000 is Y=999.97, and for the maximum observed count of highly expressed genes with N=10000 is Y=9997. To evaluate the performance of our estimator and its collision-oblivious counterpart (which estimates $\hat{N}(y)=y$), we measured their raw gene expression estimation accuracy, as well as their performance in downstream differential expression (DE) analysis.

We see the dramatic impact of collisions at shorter UMI lengths like k=5 (Figure 5a), which our method-of-moments estimator is able to correct for (Figure 5b). To aggregate this metric across genes within a cell, we normalize the gene expression vectors and compute the MSE between our estimates and the ground truth k=12 gene expression. Computed across all cells, our estimator reduced the mean squared error (MSE) by 95% over the naive estimator at a UMI length of 5 (Figure 5c). These performance improvements generalize to both additional datasets (Section S2).

Our more accurate quantification translates to improved retention of biological insights in downstream tasks. For the prototypical task of cell type prediction, we observe that even with very short UMIs (e.g. k=4), the naive estimator still allows accurate CellTypist classification [21] (over 90% accuracy, Figure S9). To generate a more meaningful comparison, we focus on differential gene expression analysis. For low-expression marker genes, presence/absence of this gene is often sufficient, and so UMI length has little effect (Figure S10). Studying more highly expressed genes, we show that our improved quantification ensures accurate estimation of their fold changes (Figure 5d,e). Aggregating the error in fold change estimation across genes highlights the improved performance of our estimator below k=8; at k=5, our estimator reduces MSE by 96% (Figure 5f).

Theoretically, our estimator yields dramatic performance improvements. We highlight these in Table 1, comparing the bias, variance, and resulting MSE of our collision-aware estimator to the naive estimator across different regimes of the number of unique transcripts N relative to the number of unique UMIs K. We assume a uniform UMI distribution for simplicity. Detailed derivations are provided in Section S7.1. Throughout, we use standard Bachmann–Landau (big-O) asymptotic notation. Our results show that in the no-collision regime $\left(N=o(\sqrt{K})\right)$, both estimators perform well. However, in the moderate-collision regime $\left(N=o(K),N=\text{Ω}(\sqrt{K})\right)$, our estimator remains unbiased with no excess variance beyond that present in Y, while the naive estimator is bias-dominated with MSE scaling as $\text{Θ}\left(N^{4}/K^{2}\right)$. In the high-collision regime (N=o(KlogK),N=Ω(K)), our estimator begins to show increasing bias and variance inflation, but still outperforms the linear in N bias of the naive estimator. Finally, in the saturation regime (N≥cKlogK for c>1), both estimators are fundamentally limited by the lack of information in Y, and exhibit linear in N bias. We further show in Sections S3 and S4 that the Poissonized maximum likelihood estimator, while theoretically optimal in the nonuniform setting, provides negligible practical improvement over the method-of-moments estimator, validating the latter as the recommended approach.

## 6 Discussion

In this work we showed that the ubiquitous approach of collapsing reads with identical UMIs as PCR duplicates is statistically biased. While conventionally the solution to this is to use longer UMIs tominimize the number of collisions, we enable the use of shorter and cheaper UMIs via a method-ofmoments estimator that adjusts for these UMI collisions. We show that our estimator is near-optimal theoretically, matching the empirical performance of the poissonized MLE, and that in practice it performs extremely well up until the threshold of saturation. This allows for robust biological and statistical conclusions to be drawn from UMIs as short as 5 bp, closely matching the results from the standard 12-bp UMIs. This has the potential to further reduce sequencing costs and simplify manual UMI synthesis, enabling more cost-effective and scalable sequencing. There are many exciting directions of future work, including incorporating error correction into our statistical model, and leveraging per-UMI counts as opposed to just the presence/absence of a given UMI.

## Supplementary Material

Supplement 1

## Figures and Tables

**Table 1:** Comparison of bias, variance, and MSE for naive and collision-aware estimators across different regimes of N. Uniform UMI distribution assumed for simplicity. Detailed derivations in Section S7.1.

_Regime_╲^Estimator^	Naive Estimator			Collision-aware Estimator		
	Bias	Variance	MSE	Bias	Variance	MSE
$N=o(\sqrt{K})$	o(1)	o(1)	o(1)	o(1)	o(1)	o(1)
N=o(K), $N=\text{Ω}(\sqrt{K})$	$\text{Θ}\left(N^{2}/K\right)$	$\text{Θ}\left(N^{2}/K\right)$	$\text{Θ}\left(N^{4}/K^{2}\right)$	o(1)	$\text{Θ}\left(N^{2}/K\right)$	$\text{Θ}\left(N^{2}/K\right)$
N=o(KlogK), N=Ω(K)	Θ(N)	$\text{Θ}\left(Ke^{-N/K}\right)$	$\text{Θ}\left(N^{2}\right)$	$\text{Θ}\left(e^{N/K}\right)$	$\text{Θ}\left(Ke^{N/K}\right)$	$\text{Θ}\left(Ke^{N/K}\right)$
N≥cKlogK for c>1	Θ(N)	o(1)	$\text{Θ}\left(N^{2}\right)$	Θ(N)	o(1)	$\text{Θ}\left(N^{2}\right)$

## Data Availability

All datasets analyzed in this study are publicly available from 10x Genomics (PBMC 1k, 5k, and 10k datasets):
1k PBMC: https://www.10xgenomics.com/datasets/1-k-pbm-cs-from-a-healthy-donor-v-3-chemistry-3-standard-3-0 10k PBMC: https://www.10xgenomics.com/datasets/10k-human-pbmcs-3-v3-1-chromium-controller-3-1-high 5k PBMC: https://www.10xgenomics.com/datasets/5k_Human_Donor1_PBMC_3p_gem-x Source code implementing the collision-aware estimator is available at: https://github.com/agdylan/minimalUMIs under the MIT License. The version used for this manuscript corresponds to commit id: 021a029.
