# Supplementary material for: Unique molecular identifiers don’t need to be unique: a collision-aware estimator for RNA-seq quantification
Source: bioRxiv. 2026 May 20:2025.09.08.674884. Preprint. [Version 2] doi: 10.1101/2025.09.08.674884 (PMC13228633; doi:10.1101/2025.09.08.674884)
Supplement: Supplement 1 [file NIHPP2025.09.08.674884v2-supplement-1.pdf]

bioRxiv preprint doi: https://doi.org/10.1101/2025.09.08.674884; this version posted May 21, 2026. The copyright holder for this preprint (which was not certified by peer review) is the author/funder, who has granted bioRxiv a license to display the preprint in perpetuity. It is made available under a CC-BY 4.0 International license.

Supplementary Information

Supplement roadmap

This supplement provides additional methodological detail, extended theoretical results, and supplementary analyses supporting the main text. Section S1 details data processing, synthetic UMI truncation, fitting the truncated-synthesis model, sequencing-error considerations, differential expression analysis details, and MALAT1 analysis details. Section S2 provides analogues of our 1k PBMC results figure (Fig. 5) on two additional PBMC datasets (10k v3 and 5k v4) to validate the generality of our method, and includes DE diagnostics. Section S3 derives the maximum likelihood estimator under a Poissonized model and analyzes its properties. Section S4 empirically validates different UMI distribution models and collision-aware estimators, showing that the method-of-moments estimator with a constant PWM captures nearly all available gains. Section S5 derives estimator variance and convexity (and the $Y = K$ extrapolation). Section S6 leverages the asymptotic normality of Y to characterize the bias and variance of our estimator. Section S7 computes and compares the MSE of our estimator and the naive estimator across different regimes of N (details for Table 1). It also proves the impossibility of estimation for $N \geq cK \log K$ for $c > 1$, and shows that our estimator matches the Cramér–Rao lower bound in a simplified binomial setting.

Supplementary Table of Contents

S1 Datasets and processing details **13**
S1.1 Synthetically truncated UMIs . . . 13
S1.2 Fitting truncated UMI synthesis model . . . 13
S1.3 Sequencing error . . . 15
S1.4 Differential Expression processing details . . . 16
S1.5 MALAT1 analysis . . . 17
S1.6 Synthetic data generation for Figure 1 . . . 17

S2 Validation on additional datasets **17**
S2.1 DE supplemental figures . . . 19

S3 Maximum Likelihood Estimator in Poissonized Setting **20**
S3.1 Sufficient statistics . . . 20
S3.2 Poissonized Setting . . . 21
S3.2.1 Method of Moments Estimator in Poissonized Setting . . . 22

S4 Empirical validation of different UMI estimates and collision-aware estimators **22**
S4.1 UMI distribution modeling . . . 23
S4.2 Empirical validation of poissonized MLE . . . 25

S5 Theoretical analysis for the method-of-moments estimator **27**
S5.1 Estimator convexity . . . 27
S5.1.1 Extension to nonuniform UMI distributions . . . 27
S5.2 Variance of MoM estimator . . . 27
S5.3 Runtime and memory analysis of Method-of-Moments Estimator . . . 28

S6 Asymptotic normality analysis **28**
S6.1 Asymptotic variance of MoM estimator . . . 29
S6.2 Asymptotic bias of MoM estimator . . . 29
S6.3 Extension to nonuniform UMIs . . . 30

S7 Optimality of method-of-moments estimator for uniform UMIs **31**
S7.1 MSE analysis and comparison . . . 31
S7.2 Impossibility beyond $N > K \log K$: proof of Proposition 2 . . . 32
S7.2.1 Extending saturation threshold beyond $K \log K$ by adjusting the UMI distribution 33
S7.3 Cramér–Rao lower bound in binomial setting . . . 33

bioRxiv preprint doi: https://doi.org/10.1101/2025.09.08.674884; this version posted May 21, 2026. The copyright holder for this preprint (which was not certified by peer review) is the author/funder, who has granted bioRxiv a license to display the preprint in perpetuity. It is made available under a CC-BY 4.0 International license.

S1 Datasets and processing details

We processed all datasets using Cell Ranger. According to Cell Ranger, the 1K PBMC (v3) dataset contains 1222 cells sequenced at an average depth of 54k reads/cell, the 10K PBMC dataset (v3) contains 11485 cells sequenced at an average depth of 30k reads/cell, and the 5k PBMC dataset (v4) contains 4782 cells sequenced at an average depth of 39k reads/cell. To synthetically generate shortened UMIs, we processed the output BAM file from Cell Ranger, and shortened the UB field (corrected UMI, discussed in Section S1.3) to the first k base pairs for UMI length k (removing the last 12-k base pairs)

For downstream analyses, for each dataset and UMI length we performed the standard pipeline of library-size (total count) normalization, scaling to 10,000 counts per cell followed by a log1p transformation. We use CellTypist [21] to annotate each cell, using their `Healthy_COVID19_PBMC` model, which is trained on "peripheral blood mononuclear cell types from healthy and COVID-19 individuals", as this is the best match for our datasets.

S1.1 Synthetically truncated UMIs

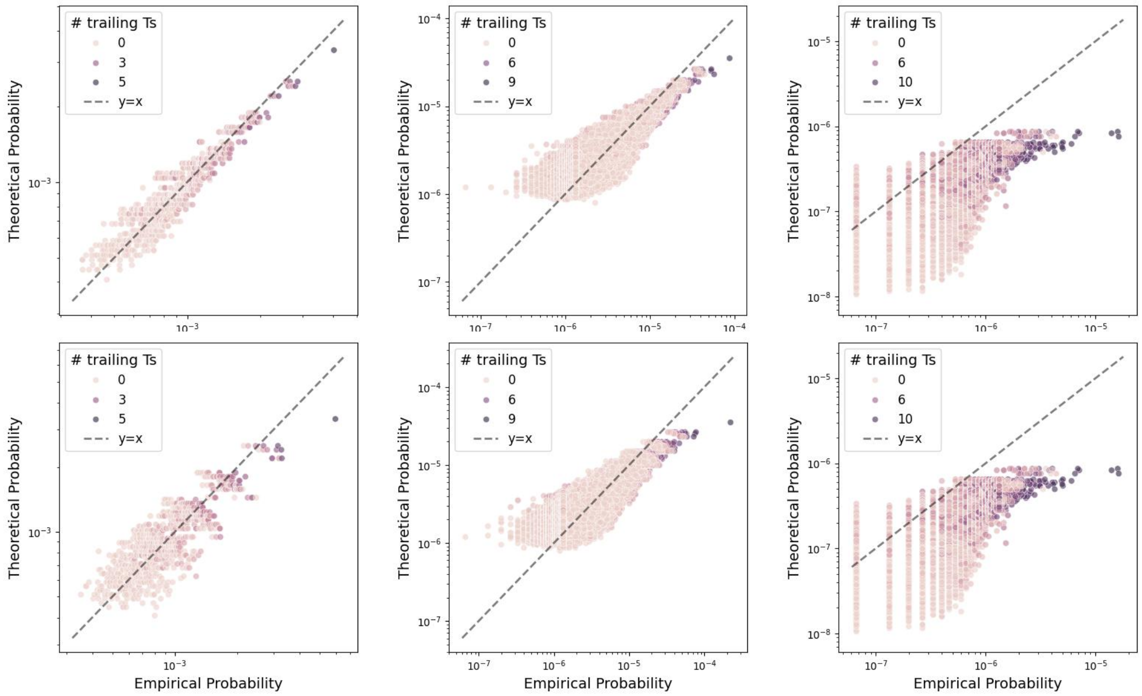

Fig. S1: **Accuracy of UMI frequency model in Equation (8).** Columns correspond to UMI lengths of 5 (left), 9 (center), and 12 (right). Top row corresponds to truncating UMIs from the rear (removing the last $12 - k$ base pairs, as we use for all datasets), and the bottom row to truncating UMIs from the front (to show our model's robustness). Note that the last column is the same for both rows, as the UMIs are full length (this plot is also included in Figure 3a). Both settings are fit by the constant PWM model reasonably well, with an improved fit when k is short.

S1.2 Fitting truncated UMI synthesis model

UMI synthesis is an error-prone procedure. Recall the truncated UMI synthesis model posited in Equation (9) for a UMI S of length k with $\texttt{T}$ trailing Ts, motivated by [14]:

$$\mathbb{P}(S) = \sum_{\ell=k-\texttt{T}}^{k} \mathbb{P}(S_{:\ell})(1-q)^{\ell} q^{\mathbb{1}\{\ell<k\}} = \sum_{\ell=k-\texttt{T}}^{k} \prod_{i=1}^{\ell} \mathbb{P}(S_i)(1-q)^{\ell} q^{\mathbb{1}\{\ell<k\}}. \tag{10}$$

This is based on the assumption that each base pair is synthesized successfully with probability $1-q$, independently across base pairs. When synthesis fails at a given base pair, the UMI is capped at that position (length $< k$), and the sequencer reads past the end of the truncated UMI into the poly(dT) tail, leading to trailing Ts in the observed UMI (shown schematically in Figure S3).

bioRxiv preprint doi: https://doi.org/10.1101/2025.09.08.674884; this version posted May 21, 2026. The copyright holder for this preprint (which was not certified by peer review) is the author/funder, who has granted bioRxiv a license to display the preprint in perpetuity. It is made available under a CC-BY 4.0 International license.

We see that this model can be extended to the case of per-bp synthesis failure probabilities q_i:

$$\mathbb{P}(S) = \sum_{\ell=k-\mathtt{T}}^{k} \mathbb{P}(S_{:\ell}) \prod_{i=1}^{\ell} (1-q_i) q_{\ell+1}^{\mathbb{1}\{\ell<k\}} = \sum_{\ell=k-\mathtt{T}}^{k} \prod_{i=1}^{\ell} \mathbb{P}(S_i)(1-q_i) q_{\ell+1}^{\mathbb{1}\{\ell<k\}}. \quad (11)$$

We fit this model for the aggregated statistics over values of $\mathtt{T}$ in the 1k PBMC dataset, using the nucleotide frequency from the first 6-bp: [.23, .25, .20, .32] (as opposed to [.23, .24, .21, .32] when computed over the full UMI). We show that fitting monotonically increasing q_i provides a good balance between flexibility and avoiding overfitting, as shown in Figure S2. The monotonically increasing q_i fitted on the 1k PBMC dataset yield good predictive performance for the 10k PBMC dataset, as shown in Figure S4.

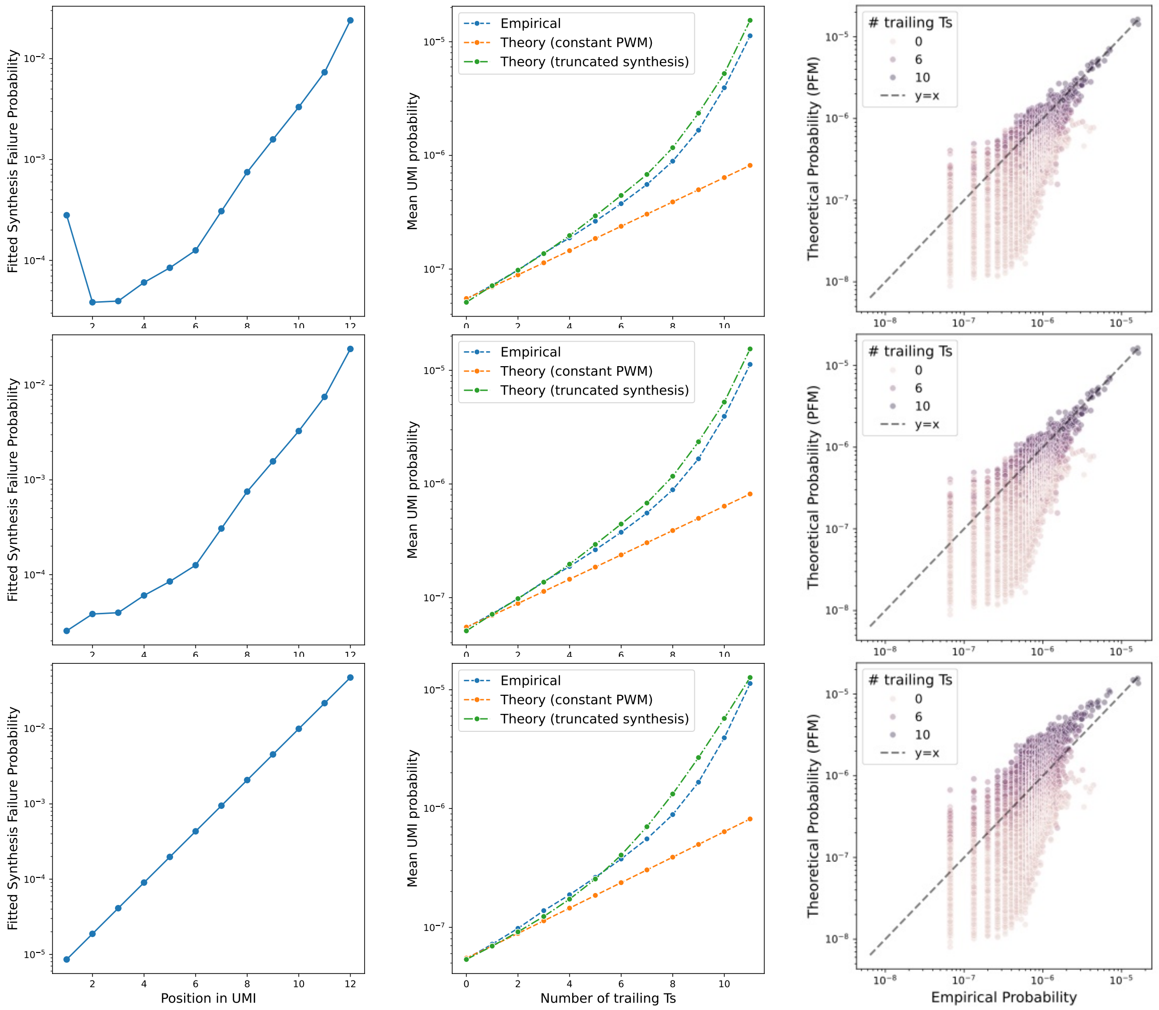

Fig. S2: **Fitting per-bp UMI synthesis failure probabilities.** We fit three models for the per-bp synthesis failure probabilities q_i, where the first row corresponds to unconstrained q_i, the second row to monotonically increasing q_i, and the third row to linearly increasing q_i in log-scale. The left column shows the fitted q_i, the center column the mean observed vs. predicted UMI probabilities grouped by the number of trailing Ts, and the right column the observed vs. predicted UMI probabilities on a per-UMI basis.

bioRxiv preprint doi: https://doi.org/10.1101/2025.09.08.674884; this version posted May 21, 2026. The copyright holder for this preprint (which was not certified by peer review) is the author/funder, who has granted bioRxiv a license to display the preprint in perpetuity. It is made available under a CC-BY 4.0 International license.

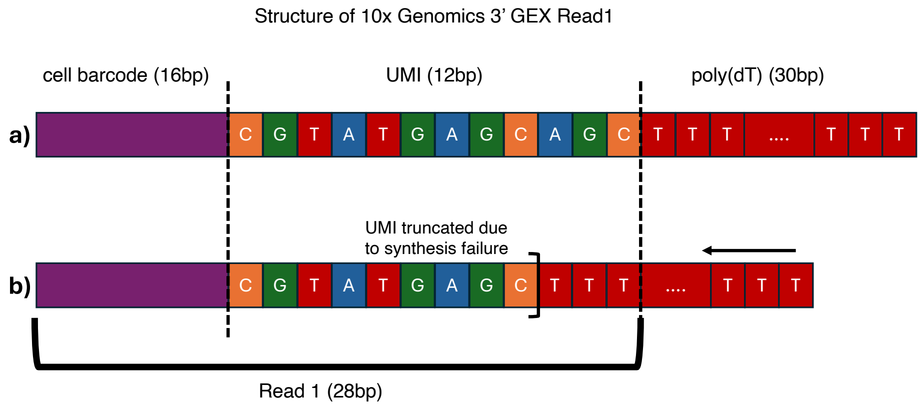

Fig. S3: **Schematic of Read1, how synthesis failure leads to trailing Ts.** During library preparation, a poly(dT) tail is synthesized immediately after the UMI. If UMI synthesis fails at a given base pair, the sequencer reads past the end of the truncated UMI into the poly(dT) tail, leading to trailing Ts in the observed UMI. **a)** shows successful synthesis of a full length UMI, where the 16bp barcode is fully present, and the 12bp UMI is fully synthesized, leading to the desired 28bp Read1. **b)** shows a UMI where synthesis fails at the 10th base pair, leading to a truncated UMI of length 9. However, the sequencer reads past the end of this truncated UMI, leading to 3 trailing Ts.

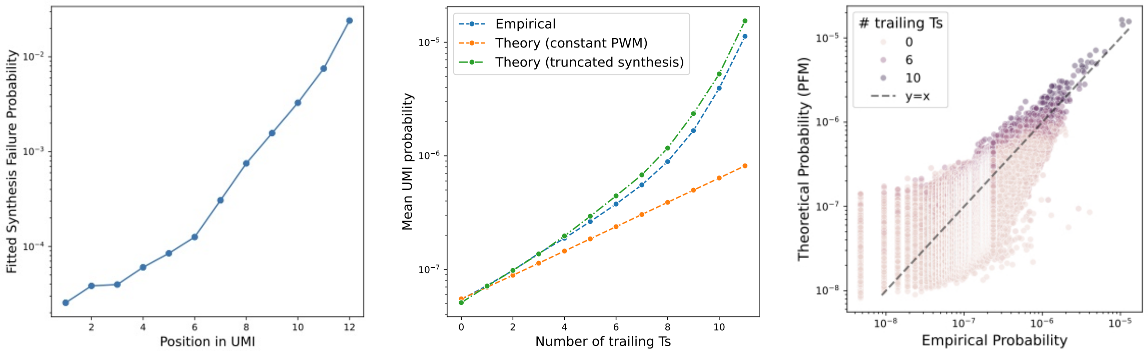

Fig. S4: **Evaluating per-bp UMI synthesis failure probabilities on 10k PBMC dataset.** We leverage the fitted monotonically increasing q_i from the 1k PBMC dataset (left) to predict UMI frequencies in the 10k PBMC dataset. The center column shows the mean observed vs. predicted UMI probabilities grouped by the number of trailing Ts, and the right column the observed vs. predicted UMI probabilities on a per-UMI basis. Due to the larger dataset size, and hence more low count UMIs observed, the fit is not as close as in the 1k PBMC dataset, but the overall trends are still captured well. A more careful fitting procedure, better taking into account the absent UMIs could yield an improved / generalizable fit.

S1.3 Sequencing error

As noted, our model does not account for UMI sequencing errors. Errors introduced during PCR amplification or during sequencing impact the actual UMIs we observe, leading to incorrectly undeduplicated counts: UMIs that should have been deduplicated but are not due to errors. Consider for concreteness the case where a gene has 100 reads associated with it, all stemming from 2 unique UMIs. However, during sequencing, one of these reads suffers a sequencing error at the last base pair of its UMI. Then, naively, the number of unique UMIs recorded as the ground truth for this gene is 3. However, for a UMI length of 11 or shorter, if we only observe the first k base pairs of the UMI, our count will now be only 2, as the errored base pair will be deleted from the UMI and there will only be the true 2 unique UMIs.

For simplicity, in this work we analyze Cell Ranger's error corrected UMIs (UB instead of UR field in BAM file), noting that these are functions of the full length UMI which we would in practice not observe. Algorithmically, UMI error correction methods utilize the fact that neighboring UMIs in Hamming space (i.e. UMIs one base pair apart) are very rare naturally for long UMIs, and so these are collapsed and considered sequencing errors. However, as the UMI length shortens, the number of

bioRxiv preprint doi: https://doi.org/10.1101/2025.09.08.674884; this version posted May 21, 2026. The copyright holder for this preprint (which was not certified by peer review) is the author/funder, who has granted bioRxiv a license to display the preprint in perpetuity. It is made available under a CC-BY 4.0 International license.

possible UMIs decreases exponentially, and so the probability of two UMIs being one base pair apart increases. In fact, a naive use of existing error correction methods, e.g. collapsing UMIs which are Hamming distance 1 and one has at least a factor of 2 plus 1 more counts than the other [11], may yield a *non-monotonic* relationship between the observed UMI counts and the true UMI counts, as the UMI space becomes so saturated that many valid UMIs are incorrectly collapsed together.

This issue of UMI error correction for short UMIs was briefly studied in [13], with a Bayesian method that worked to jointly model collisions and sequencing error, but due to the computationally intensive nature of their proposed estimator their approximate solution had to be further simplified by quantizing a dynamic programming problem. Note that any UMI error correction methods can be used as preprocessing for our algorithm, which can work to avoid such issues by utilizing information such as Phred scores, counts, and graph-based rules [10–12].

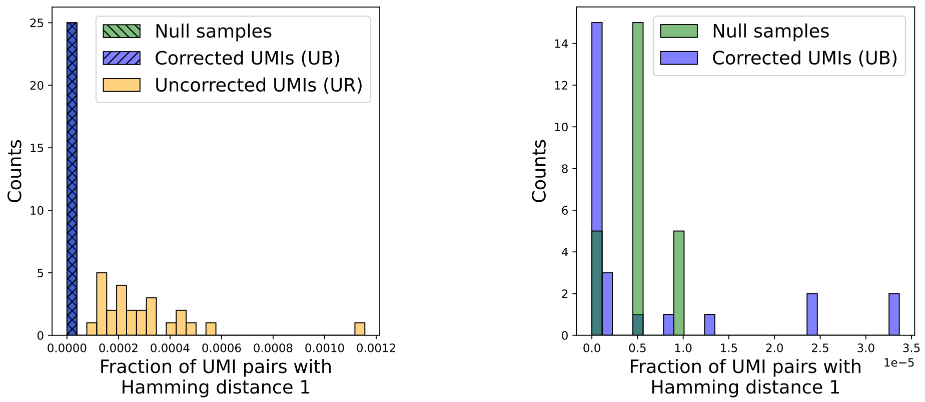

(a) (b)

Fig. S5: **Pairwise Hamming distance between UMIs shows errors in uncorrected UMIs.** We compute pairwise Hamming distances between all UMIs for a given gene in a given cell. Null samples generated by sampling n UMIs without replacement from the empirical UMI distribution across all cells all genes. We plot the top 5 genes over the top 5 cells, and choose n as the mean UB counts over these. **a)** shows all three methods, and **b)** zooms in on the corrected UMIs and null samples.

An empirical analysis of the 1k PBMC dataset reveals that many more uncorrected UMIs are 1 base pair apart than would be expected by chance (Figure S5). We study the top m cells, and their expression of the top m most expressed genes ($m = 5$). We compute, across all pairs of UMIs, what fraction are 1 base pair apart. In addition to the uncorrected (UR) and corrected (UB) UMIs, we also generate a null distribution by randomly sampling a matching number of UMIs from the empirical error corrected UMI distribution (UB) across all cells and all genes. Plotting histograms of these three, we see that the uncorrected UMIs have a significantly higher fraction of pairs that are 1 base pair apart compared to the corrected UMIs and the null distribution, separated by an order of magnitude (Figure S5a). Zooming in on the corrected UMIs and the null samples, these are statistically very similar, validating our theoretical model that UMIs within a given cell, for a given gene, are drawn i.i.d. from some common distribution across cells / genes (Figure S5b).

S1.4 Differential Expression processing details

We perform differential expression analysis to assess our estimator's ability to recover biological insights. To this end, we select cell types with over 100 cells in the 1k dataset (CD14 monocytes, Naive B cells, and Naive CD4+ T cells) and run the DE analysis pipeline as described below on each of them, and aggregate the results. For each counts matrix, after normalization and log1p transformation, we run scanpy's rank genes group function with the Wilcoxon rank sum test to compute differential expression statistics [22]. For each gene we compute the difference between the estimated and ground truth log-fold changes, using either the naive estimator or collision-aware one. An example of this is shown in Figure 5d-f.

In our analysis, we observe several marker genes for which UMIs are unnecessary for detecting differential expression. For example, we observe that FCN1 is a marker gene for CD14 monocytes, which displays essentially a binary expression pattern (Figure S10, additional discussion in Section S2.1). Simply the presence or absence of this gene (UMI length of 0) is sufficient. Such genes are not of

bioRxiv preprint doi: https://doi.org/10.1101/2025.09.08.674884; this version posted May 21, 2026. The copyright holder for this preprint (which was not certified by peer review) is the author/funder, who has granted bioRxiv a license to display the preprint in perpetuity. It is made available under a CC-BY 4.0 International license.

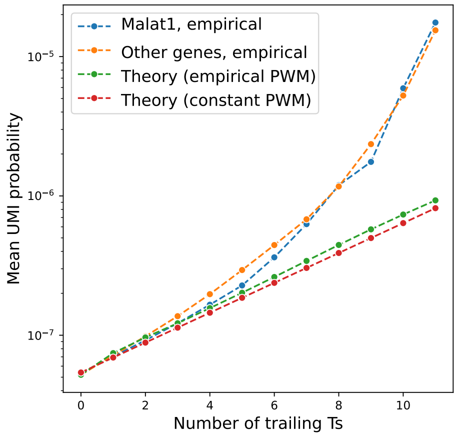

Fig. S6: **Analysis of empirical UMI nucleotide frequencies in 1k PBMC dataset.** Mean probability for all UMIs given a certain number of trailing Ts. This is shown for the empirical frequencies of MALAT1 (blue) and all other genes (orange), as well as the theoretical probabilities based on the PWM (subfigure **a)** , green) and the constant PWM (Equation (8), red).

interest to study here, as UMIs are not even needed in the first place. To this end, we filter for genes with mean expression greater than 10 across all cells in the dataset. Additionally, similar to a volcano plot, we filter for genes with at least a 25% fold change in either direction, with an adjusted p-value less than 0.05.

S1.5 MALAT1 analysis

Here, we provide the details for the UMI-based identification that MALAT1 is an outlier (shown in Figure 4) We can approximate the variance of the TV distance for m counts by noting that under the model that the nucleotides of a UMI are independent and identically distributed, we are computing the variance of the TV distance between a sample of size m from a multinomial and its expectation. For large m, the entries of the multinomial are approximately independent. Concretely, denoting $\boldsymbol{p}$ as the nucleotide distribution, and $X \sim \text{Multinomial}(m, \boldsymbol{p})$:

$$\text{Var}\left(\text{TV}(X/m, \boldsymbol{p})\right) = \text{Var}\left(\frac{1}{2}\sum_{i=1}^{4}\left|\frac{X_i}{m} - p_i\right|\right) \approx \frac{1}{4}\sum_{i=1}^{4}\text{Var}\left(\frac{X_i}{m}\right) = \frac{1}{4m}\sum_{i=1}^{4} p_i(1-p_i) \qquad (12)$$

S1.6 Synthetic data generation for Figure 1

For Figure 1, we generate synthetic data to show the natural behavior when UMIs are long (Figure 1b), when they are short and yield collisions (Figure 1d), and how we can correct for this by utilizing our method-of-moments estimator (Figure 1e). To generate the synthetic UMI data, we simulated the labeling process under a uniform distribution. For a given UMI length k, UMIs are simulated as being drawn uniformly at random with replacement from all $K = 4^k$ possible sequences. To model a true transcript abundance of N, we take N independent draws from this pool and record the resulting number of unique UMIs observed as Y. This process was evaluated across logarithmically spaced values of N to capture the system's behavior from a low-collision regime up through complete saturation.

S2 Validation on additional datasets

Throughout, we discussed the application of our method to 10x's PBMC 1k dataset. Here, we show that our method's performance improvements hold in general. We recapitulate our analyses from Figure 5 for the 10x Genomics 10k PBMC dataset with v3 chemistry (Figure S7), and for the 10x Genomics 5k PBMC dataset with v4 chemistry (Figure S8).

bioRxiv preprint doi: https://doi.org/10.1101/2025.09.08.674884; this version posted May 21, 2026. The copyright holder for this preprint (which was not certified by peer review) is the author/funder, who has granted bioRxiv a license to display the preprint in perpetuity. It is made available under a CC-BY 4.0 International license.

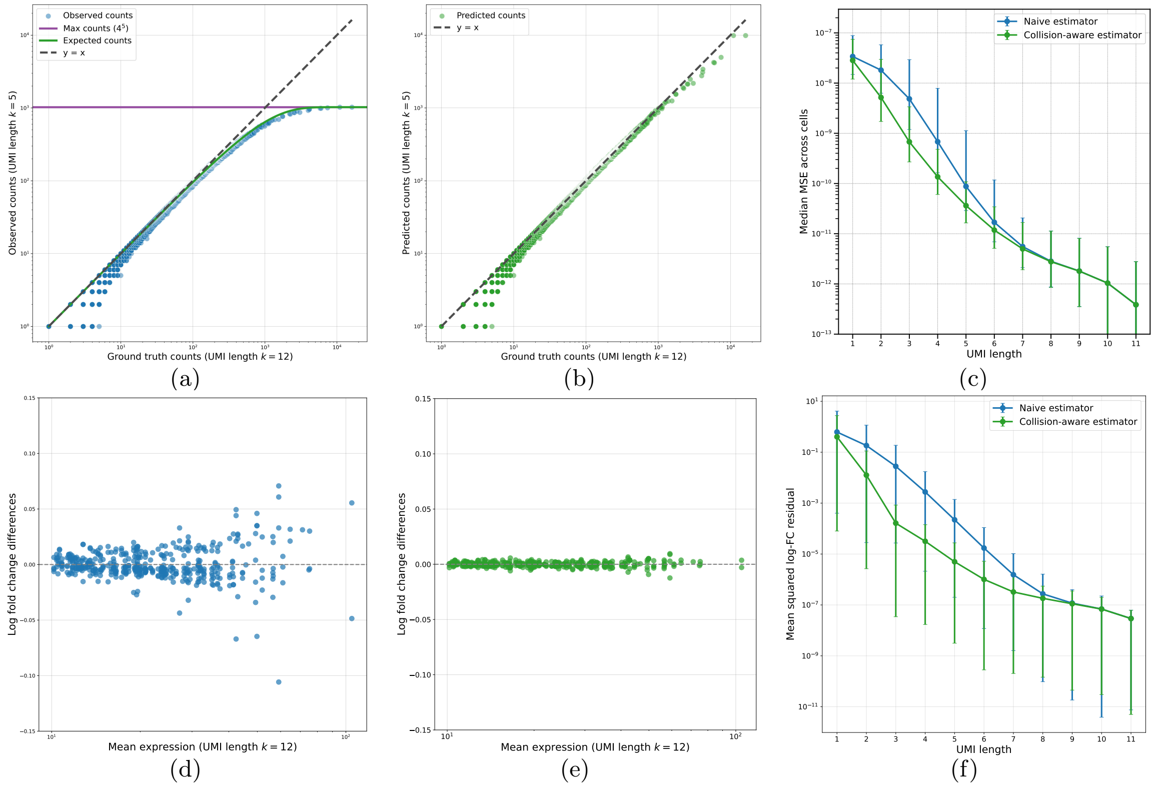

Fig. S7: **Performance improvement afforded by our method-of-moments estimator on 10x's PBMC 10K dataset (v3 chemistry). a-c** show improvement in raw expression estimation, and **d-f** show improvement for log-fold change (LFC) estimation of differentially expressed genes, replication of Figure 5.

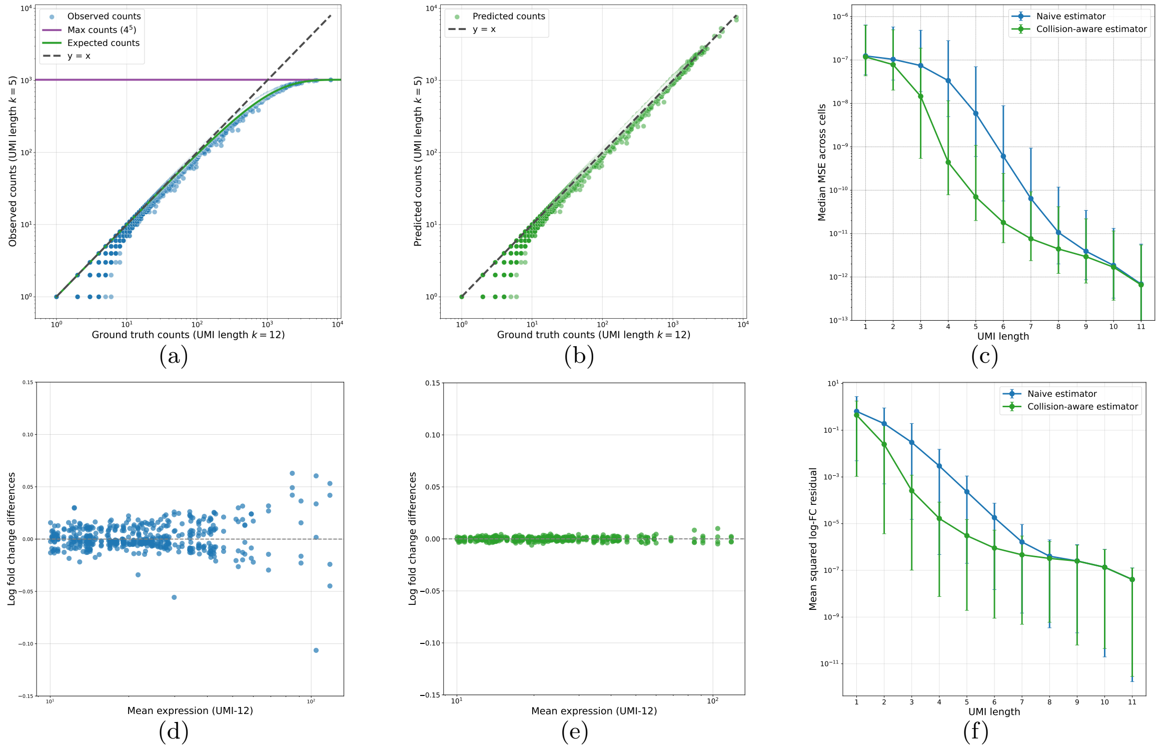

Fig. S8: **Performance improvement afforded by our method-of-moments estimator on 10x's PBMC 5K dataset (v4 chemistry). a-c** show improvement in raw expression estimation, and **d-f** show improvement for log-fold change (LFC) estimation of differentially expressed genes, replication of Figure 5.

bioRxiv preprint doi: https://doi.org/10.1101/2025.09.08.674884; this version posted May 21, 2026. The copyright holder for this preprint (which was not certified by peer review) is the author/funder, who has granted bioRxiv a license to display the preprint in perpetuity. It is made available under a CC-BY 4.0 International license.

S2.1 DE supplemental figures

To begin, in Figure S9, we highlight the ease of cell type annotation as a computational task. Even for very short UMI lengths like $k = 4$, we still retain essentially the same accuracy with the naive estimator as for $k = 9$, showing the cell type annotation is insufficiently sensitive to actual counts (and by extension, UMI length) to serve as our benchmark.

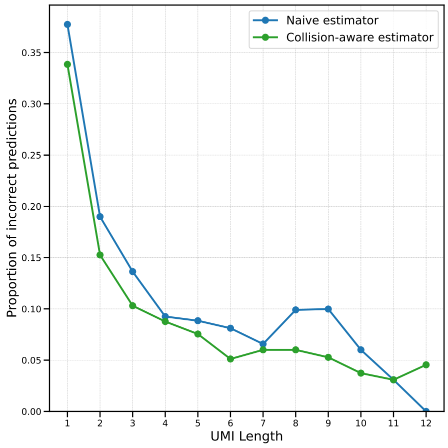

Fig. S9: Fraction of errors in cell type prediction using CellTypist as a function of UMI length.

As discussed in Section S1.4, we filter out low count differently expressed genes from our analysis. This is because, for certain marker genes that exhibit a binary-like expression (0 in certain cells, and nonzero in others), we don't actually need UMIs *at all* to detect that this gene is differentially expressed. FCN1, a marker gene for CD14 Monocytes, overwhelmingly displays 0 expression for other cell types, with only 12% of CD14 monocytes having 0 expression, while over 93% of other cells have 0 counts (Figure S10). With a UMI length of 12, the LFC is 5.9 with a p-value of 1.1×10^{-118}, while with a UMI length of 0 the naive estimator provides an LFC of 4.2 with a p-value of 2.5×10^{-110}. Clearly, an improved estimator is unnecessary in this situation, and so we filter out genes with low average expression to ensure that we are only comparing those where UMI length will play a role.

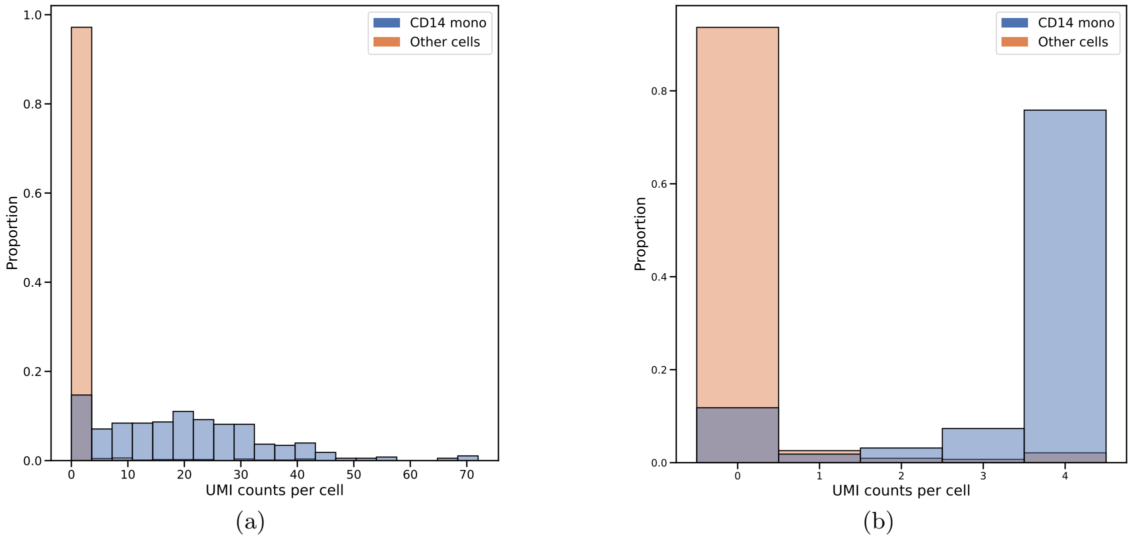

Fig. S10: **Marker genes do not require UMIs to be called as DE.** Marker genes that display a binary expression pattern are called as differentially expressed even by the naive estimator for a UMI length of 1. Shown is FCN1, a marker gene for CD14 monocytes. **a)** Distribution of raw UMI counts for the gene FCN1 analyzing the full length 12 UMIs. **b)** Same as **a** but using the raw length 1 UMI counts (the naive estimator).

bioRxiv preprint doi: https://doi.org/10.1101/2025.09.08.674884; this version posted May 21, 2026. The copyright holder for this preprint (which was not certified by peer review) is the author/funder, who has granted bioRxiv a license to display the preprint in perpetuity. It is made available under a CC-BY 4.0 International license.

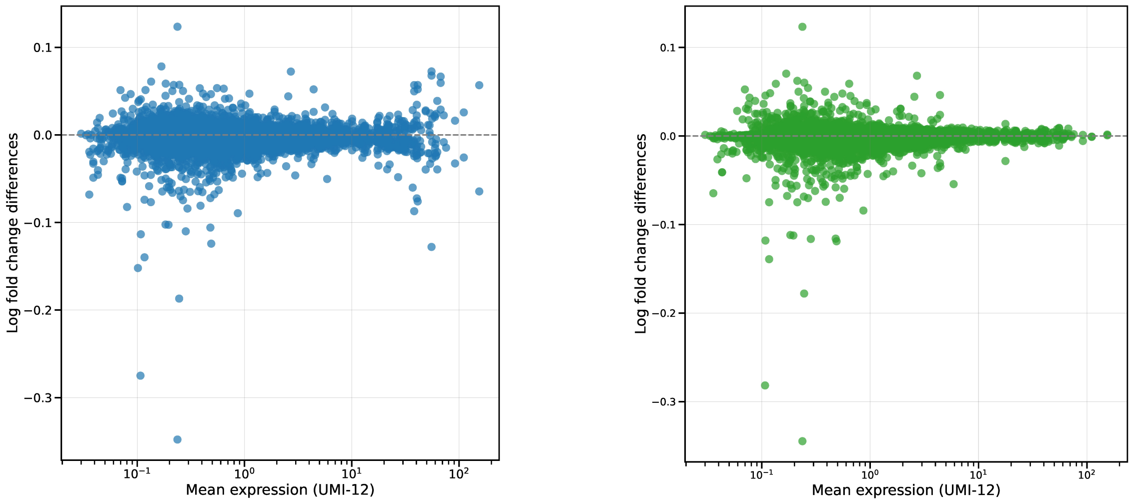

(a) (b)

Fig. S11: **Mean expression versus LFC for all DE genes (no average expression filter) for 1k PBMC dataset.** Difference between true LFC and predicted LFC using naive estimator and method-of-moments estimator for a 5-bp UMI. We filter for genes with significant p-value and moderate fold change. Performance is similar for the two methods at low expression levels, as these genes often do not even require the use of UMIs (Figure S10). As mean expression increases however, our estimator shows its improved performance (highlighted in Figure 5d,e). **a)** Log-fold changes between ground truth and naive estimator for all genes. **b)** Same as **a** but for our collision-aware estimator.

S3 Maximum Likelihood Estimator in Poissonized Setting

Recall our statistical model: N identical balls are randomly assigned into K bins, with the observation Y denoting the number of bins with at least one ball. In our sequencing model, this translates to N mRNA transcripts before PCR amplification, $K = 4^k$ possible UMIs, and Y unique UMIs observed. The balls (transcripts) follow some distribution p over the set $[K] = \{1, 2, \dots, K\}$, and are independent and identically distributed. Then, recall Equations (1) and (2):

$$X_1, X_2, \dots, X_N \overset{\text{i.i.d.}}{\sim} \boldsymbol{p}, \tag{13}$$

$$Y = \#\ \text{unique}\{X_1, X_2, \dots, X_N\}. \tag{14}$$

Mathematically, $Y = |\{X_1, X_2, \dots, X_N\}|$, where $|S|$ denotes the cardinality of a set S, in our case the number of distinct UMIs observed. Given p and Y, we want to estimate N. By representing Y as a sum of K indicators, we can compute its mean as in Equation (3)

$$\mathbb{E}[Y] = K - \sum_{j=1}^{K} (1 - p_j)^N \approx K - \sum_{j=1}^{K} e^{-p_j N}, \tag{15}$$

where $\hat{N}$ is computed as a method-of-moments estimator by inverting the above relationship.

S3.1 Sufficient statistics

Here, we note that we do not simply observe Y, but in fact observe some vector $W \in \mathbb{N}^K$, where W_j denotes the number of reads assigned to UMI j (after PCR amplification for each X_i). However, since we do not have a good model for PCR amplification, we ignore this information and instead only consider the signature Z of W, which is

$$Z \in \{0,1\}^K \text{ where } Z_j = \mathbb{1}\left\{\bigcup_{i=1}^{N} \{X_i = j\}\right\}. \tag{16}$$

bioRxiv preprint doi: https://doi.org/10.1101/2025.09.08.674884; this version posted May 21, 2026. The copyright holder for this preprint (which was not certified by peer review) is the author/funder, who has granted bioRxiv a license to display the preprint in perpetuity. It is made available under a CC-BY 4.0 International license.

Note that $Y = \sum_{j=1}^{K} Z_j$. However, when p is not uniform, Y is not a sufficient statistic for N. The likelihood of our observations depends on the full vector Z, not just its sum Y.

Intuition: Consider the case where we only have 2 bins ($K = 2$), where a ball is thrown into the first one with $p_1 = 0.99$, and the second with $p_2 = 0.01$. Then, if we observe occupancy $[0, 1]$, we are *reasonably* confident that this means that $N = 1$, as: $\mathbb{P}(Z = [0, 1]|N = 1) = 0.01$, while $\mathbb{P}(Z = [0, 1]|N = 2) = .01^2$. However, if we observe $Z = [1, 0]$, then our guess for N should be much larger, since it is likely that this consists of many balls all going into the first bin. Concretely, $\mathbb{P}(Z = [1, 0]|N = 1) = 0.99$, while $\mathbb{P}(Z = [1, 0]|N = 10) = 0.99^{10} \approx 0.904$, i.e. the likelihood decreases much more slowly as N increases. Formally, writing out the likelihood shows that it does not factor into a function of Y and a function independent of N, but rather depends on the full vector Z.

S3.2 Poissonized Setting

Analyzing the original model is difficult, since the indicators Z_j are dependent. To simplify the analysis, we consider the Poissonized model where instead of N balls, we have $N' \sim \text{Poisson}(N)$ balls. This is a common algorithmic analysis technique, and is a good approximation when N is large. Then, the number of balls in each bin j is independent and distributed as $\text{Poisson}(p_j N)$, due to Poisson thinning. In this case, the indicators Z_j are independent Bernoulli random variables with

$$Z_j \sim \text{Bernoulli}\left(1 - e^{-p_j N}\right). \tag{17}$$

Then, the likelihood is

$$\begin{aligned}
\mathbb{P}(Z = z) &= \prod_{j=1}^{K} \left(1 - e^{-p_j N}\right)^{z_j} \left(e^{-p_j N}\right)^{1-z_j} \\
\log \mathbb{P}(Z = z) &= \sum_{j=1}^{K} z_j \log\left(1 - e^{-p_j N}\right) + (1 - z_j)(-p_j N) \\
&= \sum_{j=1}^{K} z_j \log\left(1 - e^{-p_j N}\right) - \sum_{j=1}^{K} (1 - z_j) p_j N \\
&= \sum_{j:z_j=1} \left(\log\left(1 - e^{-p_j N}\right) + p_j N\right) - N
\end{aligned}$$

We can minimize the negative log-likelihood to obtain the MLE for N (gradient descent for a single parameter). Standard results show that the MLE is consistent and asymptotically normal, with asymptotic variance given by the inverse Fisher information. The Fisher information is (after checking the necessary regularity conditions):

$$I(N) = -\mathbb{E}\left[\frac{\partial^2}{\partial N^2} \log \mathbb{P}(Z; N)\right].$$

First, we compute the score function:

$$\begin{aligned}
\frac{\partial}{\partial N} \log \mathbb{P}(Z; N) &= \sum_{j=1}^{K} z_j \frac{p_j e^{-p_j N}}{1 - e^{-p_j N}} - \sum_{j=1}^{K} (1 - z_j) p_j \\
\frac{\partial^2}{\partial N^2} \log \mathbb{P}(Z; N) &= -\sum_{j=1}^{K} z_j \frac{p_j^2 e^{-p_j N}}{(1 - e^{-p_j N})^2}.
\end{aligned}$$

Since $\mathbb{E}[Z_j] = 1 - e^{-p_j N}$, the Fisher information is

$$I(N) = \mathbb{E}\left[\sum_{j=1}^{K} Z_j \frac{p_j^2 e^{-p_j N}}{(1 - e^{-p_j N})^2}\right]$$

bioRxiv preprint doi: https://doi.org/10.1101/2025.09.08.674884; this version posted May 21, 2026. The copyright holder for this preprint (which was not certified by peer review) is the author/funder, who has granted bioRxiv a license to display the preprint in perpetuity. It is made available under a CC-BY 4.0 International license.

$$= \sum_{j=1}^{K} (1 - e^{-p_j N}) \frac{p_j^2 e^{-p_j N}}{(1 - e^{-p_j N})^2}$$

$$= \sum_{j=1}^{K} \frac{p_j^2 e^{-p_j N}}{1 - e^{-p_j N}}. \tag{18}$$

Thus, if we compute the MLE $\hat{N}_{MLE}$ by minimizing the negative log-likelihood, we have that $\hat{N}_{MLE}$ is asymptotically normal with mean N and variance approximately equal to the inverse of the Fisher information:

$$\text{Var}(\hat{N}_{MLE}) \approx 1/I(N) = \left(\sum_{j=1}^{K} \frac{p_j^2 e^{-p_j N}}{1 - e^{-p_j N}} \right)^{-1}. \tag{19}$$

S3.2.1 Method of Moments Estimator in Poissonized Setting

In the Poissonized setting, the crossterm in the variance of Y vanishes, since the Z_j are independent. Thus, we have

$$\text{Var}(\hat{N}_{MoM}(Y)) \approx \left(\hat{N}'(Y)\right)^2 \text{Var}(Y) \approx \left(\frac{1}{f'(N)}\right)^2 \text{Var}(Y)$$
$$\approx \frac{\sum_{j=1}^{K} e^{-p_j N} \left(1 - e^{-p_j N}\right)}{\left(\sum_{j=1}^{K} p_j e^{-p_j N}\right)^2} \tag{20}$$

We can compare the asymptotic variances of the MLE and MoM estimators in the Poissonized setting. We show that $\text{Var}(\hat{N}_{MoM}) \geq \text{Var}(\hat{N}_{MLE})$, i.e., the MLE is always at least as efficient as MoM. This is equivalent to showing that

$$\frac{\sum_{j=1}^{K} e^{-p_j N}(1 - e^{-p_j N})}{\left(\sum_{j=1}^{K} p_j e^{-p_j N}\right)^2} \geq \left(\sum_{j=1}^{K} \frac{p_j^2 e^{-p_j N}}{1 - e^{-p_j N}} \right)^{-1}, \tag{21}$$

i.e., that

$$\left(\sum_{j=1}^{K} e^{-p_j N}(1 - e^{-p_j N}) \right) \cdot \left(\sum_{j=1}^{K} \frac{p_j^2 e^{-p_j N}}{1 - e^{-p_j N}} \right) \geq \left(\sum_{j=1}^{K} p_j e^{-p_j N} \right)^2. \tag{22}$$

Let $a_j = e^{-p_j N}$ and $b_j = 1 - e^{-p_j N}$. We apply the Cauchy-Schwarz inequality with

$$x_j = \sqrt{a_j b_j}, \qquad y_j = p_j \sqrt{\frac{a_j}{b_j}}.$$

Then $x_j^2 = a_j b_j$, $y_j^2 = \frac{p_j^2 a_j}{b_j}$, and $x_j y_j = p_j a_j$. By Cauchy-Schwarz, $\left(\sum_j x_j^2\right)\left(\sum_j y_j^2\right) \geq \left(\sum_j x_j y_j\right)^2$, which gives exactly (22).

Equality holds when x_j / y_j is constant across all j, i.e., when $\frac{b_j}{p_j} = \frac{1 - e^{-p_j N}}{p_j}$ is constant. This happens if and only if p_j is constant across all j, i.e., when $\boldsymbol{p}$ is uniform, validating the observation that when $\boldsymbol{p}$ is uniform, Y is a sufficient statistic and the MoM estimator is efficient. When $\boldsymbol{p}$ is not uniform, the MLE outperforms the MoM estimator, with performance gap increasing as $\boldsymbol{p}$ becomes more skewed.

S4 Empirical validation of different UMI estimates and collision-aware estimators

In this work, we have proposed degrees of estimator complexity, ranging from no UMI collision correction, to our method-of-moments estimator, to the poissonized MLE. The latter two depend on the estimated UMI distribution, which we provide models of increasing fidelity for: uniform distribution,

bioRxiv preprint doi: https://doi.org/10.1101/2025.09.08.674884; this version posted May 21, 2026. The copyright holder for this preprint (which was not certified by peer review) is the author/funder, who has granted bioRxiv a license to display the preprint in perpetuity. It is made available under a CC-BY 4.0 International license.

constant PWM (cPWM), and general PWM, the latter two optionally incorporating our synthesis failure model (monotonic synthesis failure probabilities used). Additionally, the full empirical UMI distribution can be used. We discuss these two axes of improvement separately, and show that while transcript abundance estimation improves as our estimator or UMI model gets more complex, almost all the gains can be obtained by using our method-of-moments estimator with the constant PWM.

S4.1 UMI distribution modeling

We begin by showing in Figure S12 the accuracy of different UMI distribution models, of increasing complexity, for fixed $k = 5$. We compute the L1 distance between the empirical UMI distribution (summed across all genes) and the model UMI distribution, for the 1k PBMC dataset. We analyze this for $k = 5$ since for larger k, many UMIs are not observed at all, and so the L1 distance is dominated by the large number of UMIs with 0 empirical probability. Next, we generate a scatter plot for each of these methods for $k = 12$ (Figure S13), where each point corresponds to a UMI, with x-coordinate given by the empirical probability of that UMI, and y-coordinate given by the probability of that UMI under the model. This shows how accounting for synthesis failure yields improved accuracy for the high probability UMIs with many trailing Ts.

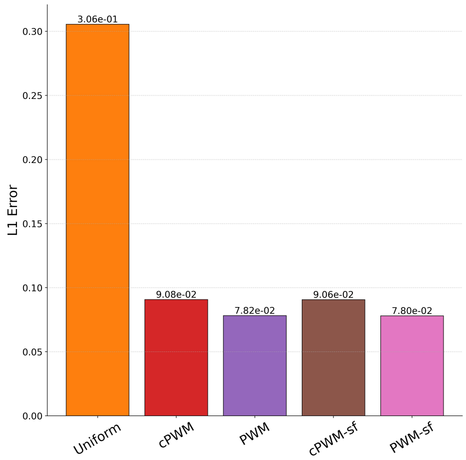

Fig. S12: Accuracy of different UMI distribution models. We compare the accuracy of different UMI distribution models, of increasing complexity, for $k = 5$. Uniform is the simplest and performs poorly, but there are extremely diminishing returns as we increase the complexity of our UMI distribution model, with the constant PWM performing almost as well as the general PWM incorporating synthesis failure.

bioRxiv preprint doi: https://doi.org/10.1101/2025.09.08.674884; this version posted May 21, 2026. The copyright holder for this preprint (which was not certified by peer review) is the author/funder, who has granted bioRxiv a license to display the preprint in perpetuity. It is made available under a CC-BY 4.0 International license.

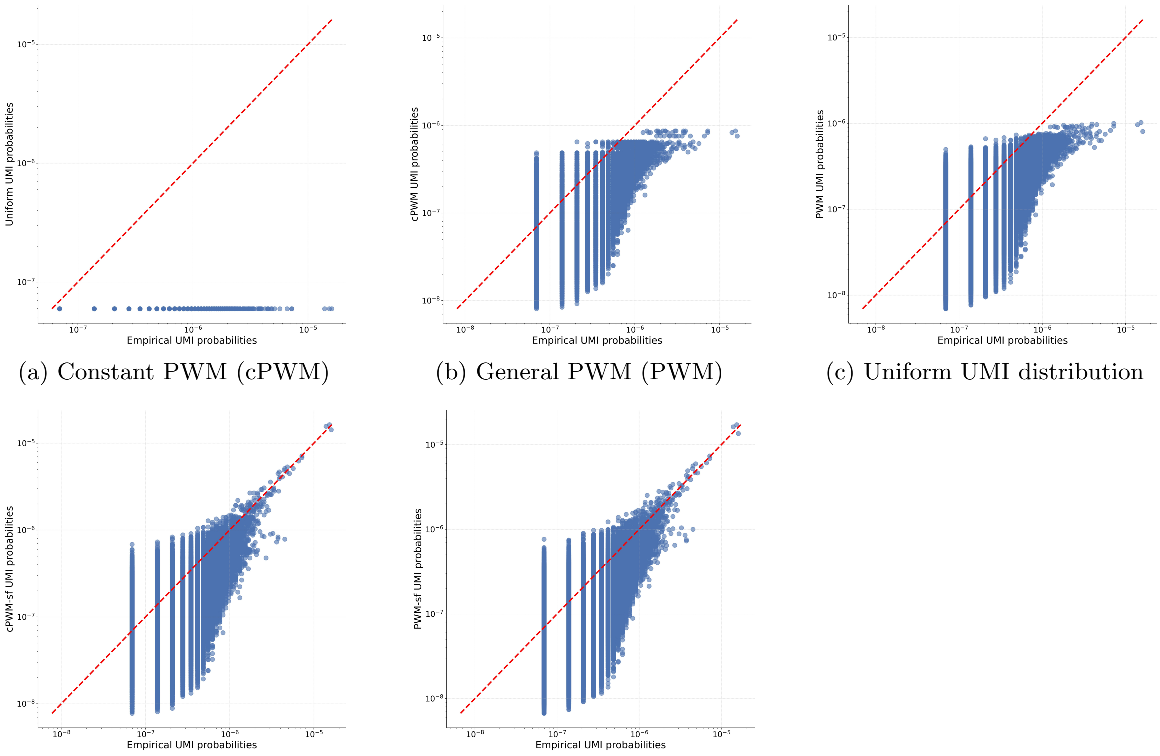

(a) Constant PWM (cPWM)
(b) General PWM (PWM)
(c) Uniform UMI distribution
(d) Constant PWM with synthesis failure (cPWM-sf)
(e) General PWM with synthesis failure (PWM-sf)

Fig. S13: UMI modeling accuracy for PBMC 1k dataset for $k = 12$, summed across all genes. Each point corresponds to a UMI, with x-coordinate given by the empirical probability of that UMI, and y-coordinate given by the probability of that UMI under the model. The uniform model clearly performs poorly,

bioRxiv preprint doi: https://doi.org/10.1101/2025.09.08.674884; this version posted May 21, 2026. The copyright holder for this preprint (which was not certified by peer review) is the author/funder, who has granted bioRxiv a license to display the preprint in perpetuity. It is made available under a CC-BY 4.0 International license.

S4.2 Empirical validation of poissonized MLE

We now study the interplay between the estimator and UMI distribution model complexity, by comparing the performance of the MLE and MoM estimators across different UMI distribution models (Figure S14). We see that the naive estimator performs very poorly for $k = 5$, with dramatic improvements afforded by the MoM estimator. However, the MLE only provides a very minor improvement over the MoM estimator, and almost all of the gains can be obtained by using the MoM estimator with the constant PWM. We show the performance per cell, gene, in Figure S15.

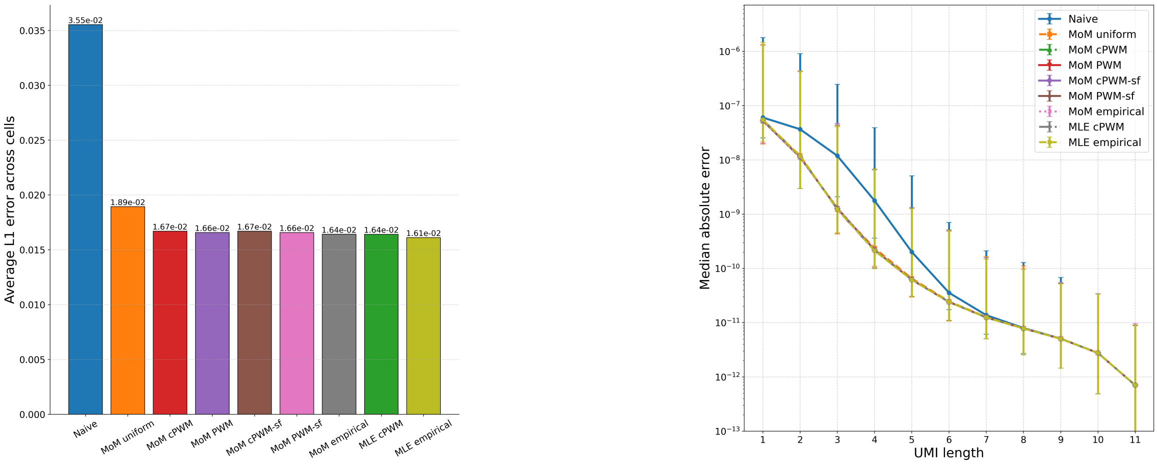

(a) Accuracy for $k = 5$.

(b) Accuracy across UMI lengths.

Fig. S14: Accuracy of different estimators (naive, method-of-moments, and MLE) across different UMI distribution models.

bioRxiv preprint doi: https://doi.org/10.1101/2025.09.08.674884; this version posted May 21, 2026. The copyright holder for this preprint (which was not certified by peer review) is the author/funder, who has granted bioRxiv a license to display the preprint in perpetuity. It is made available under a CC-BY 4.0 International license.

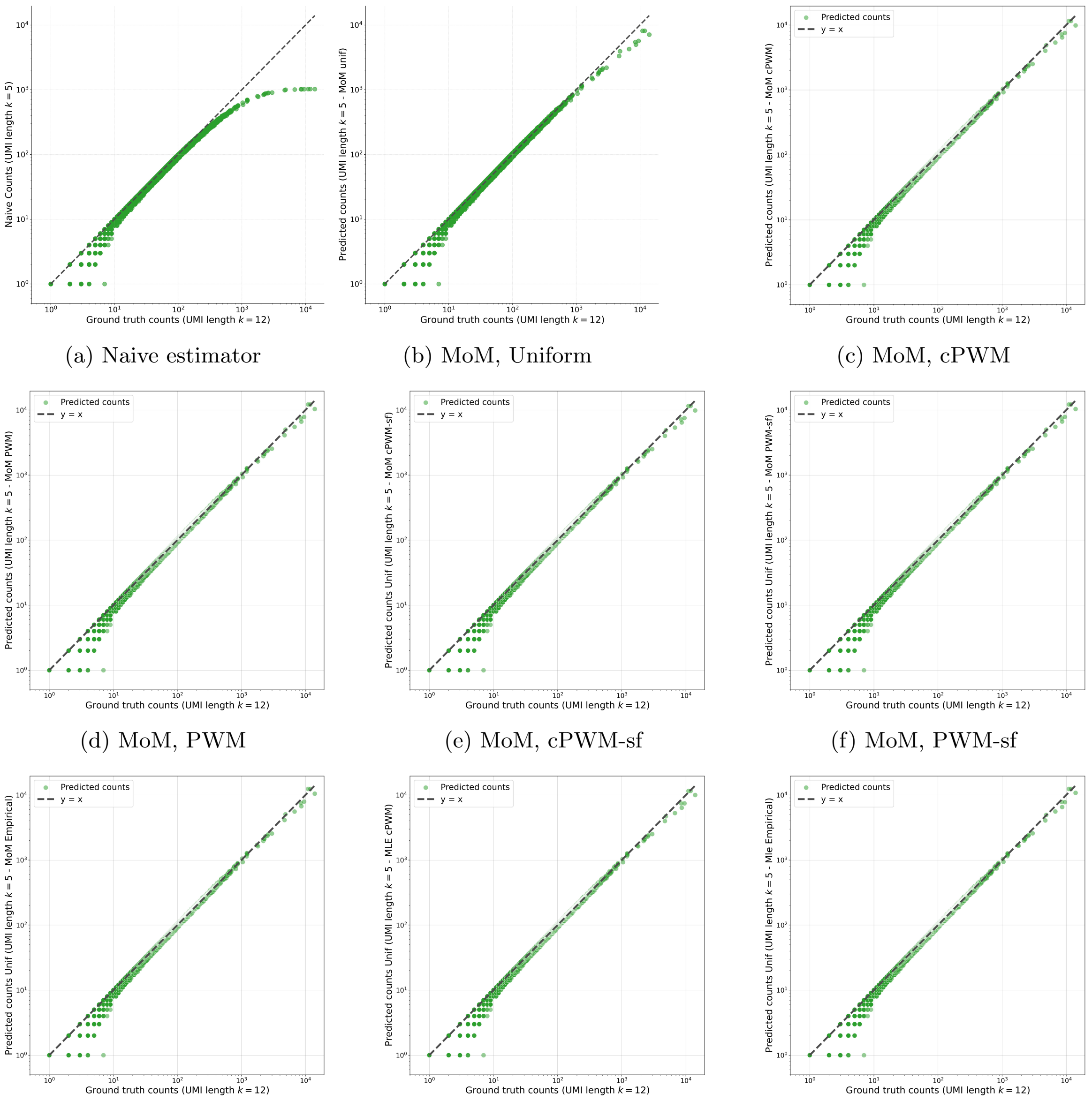

(a) Naive estimator (b) MoM, Uniform (c) MoM, cPWM

(d) MoM, PWM (e) MoM, cPWM-sf (f) MoM, PWM-sf

(g) MoM, Empirical (h) MLE, cPWM (i) MLE, Empirical

Fig. S15: Each panel shows predicted vs. ground-truth counts for a specific estimator×UMI model combination at $k = 5$. Dramatic gains by accounting for collisions, then by using a nonuniform UMI distribution. Minimal gains from using the MLE over the MoM estimator, and from using more complex UMI distribution models.

bioRxiv preprint doi: https://doi.org/10.1101/2025.09.08.674884; this version posted May 21, 2026. The copyright holder for this preprint (which was not certified by peer review) is the author/funder, who has granted bioRxiv a license to display the preprint in perpetuity. It is made available under a CC-BY 4.0 International license.

S5 Theoretical analysis for the method-of-moments estimator

In this section we provide additional theoretical results regarding our method-of-moments estimator, and give all deferred proofs.

S5.1 Estimator convexity

Proposition 1. *$\hat{N}(y)$ is convex.*

Proof For $Y < K$, $\hat{N}(Y)$ is a convex function of Y, as it is $-\log(\cdot)$ composed with an affine function. Examining the edge case of $Y = K$, we show that the derivative is strictly increasing, in that:

$$\begin{aligned}\hat{N}(K) - \hat{N}(K-1) &> \hat{N}(K-1) - \hat{N}(K-2)\\ K &> \frac{\log(1/K)}{\log(1-1/K)} - \frac{\log(2/K)}{\log(1-1/K)}\\ (1-1/K)^K &< 1/2\end{aligned} \tag{23}$$

This holds true for all $K > 1$, and so the estimator is convex. This implies that defining $\hat{N}(K) = \hat{N}(K-1)+\gamma K$ would retain the convexity of the estimator for any $\gamma > \ln(2)$. □

S5.1.1 Extension to nonuniform UMI distributions

As before, the case of $y = K$ is a priori undefined, as $Y_n < K$ for all n. Here, we use a quadratic extrapolation, to yield a simple estimate that retains the convexity of our estimator. Quadratic extrapolation here studies finite differences, i.e. the behavior of $\Delta\hat{N}(y) = \hat{N}(y) - \hat{N}(y-1)$. To obtain our quadratic extrapolation, we analyze the second finite difference $\Delta^2\hat{N}(y) = \Delta\hat{N}(y) - \Delta\hat{N}(y-1) = \hat{N}(y) - 2\hat{N}(y-1) + \hat{N}(y-2)$. To perform quadratic extrapolation, we want $\Delta^2\hat{N}(K) = \Delta^2\hat{N}(K-1)$. This simplifies as:

$$\begin{aligned}\Delta^2\hat{N}(K) &= \Delta^2\hat{N}(K-1)\\ \hat{N}(K) - 2\hat{N}(K-1) + \hat{N}(K-2) &= \hat{N}(K-1) - 2\hat{N}(K-2) + \hat{N}(K-3)\\ \hat{N}(K) &= 3\hat{N}(K-1) - 3\hat{N}(K-2) + \hat{N}(K-3)\end{aligned} \tag{24}$$

This retains the convexity of our estimator, even in the nonuniform p setting, as can be verified by computing that $\Delta\hat{N}(K) \geq \Delta\hat{N}(K-1)$.

S5.2 Variance of MoM estimator

We compute the variance of our estimator $\hat{N}(y)$ using the delta method, a first order Taylor Expansion. Let $f(N) = Y_N$ be the function relating the true number of transcripts N to the observed count $Y_N = \mathbb{E}[Y]$. Recall that

$$f(N) = K - \sum_{j=1}^{K} (1-p_j)^N \approx K - \sum_{j=1}^{K} e^{-p_j N}$$

$$f'(N) = -\sum_{j=1}^{K} \log(1-p_j)\,(1-p_j)^{N-1} \approx \sum_{j=1}^{K} p_j e^{-p_j N}$$

where in the last line we used that $\log(1+x) \approx x$ for small x, $(1-x)^N \approx e^{-xN}$ for small x, and that N is large. Using the delta method, we evaluate $\hat{N}'(y)$, the derivative of the inverse function, evaluated at the observed count y. Noting that $\hat{N}(Y) = f^{-1}(Y)$ up to the linear interpolation we perform, $\frac{d}{dy}f^{-1}(Y) = \frac{1}{f'(f^{-1}(Y))} = \frac{1}{f'(\hat{N}(Y))} \approx \frac{1}{f'(N)}$, where by construction of our estimator $\hat{N}(Y) \approx N$. In the uniform case, where $p_j = \frac{1}{K}$ for all j, this simplifies as $f'(N) \approx \sum_{j=1}^{K} p_j e^{-p_j N} = e^{-\frac{N}{K}}$. The variance of Y is given in Equation (4), and evaluating for general p yields:

$$\mathrm{Var}(\hat{N}(Y)) \approx \left(\hat{N}'(Y)\right)^2 \mathrm{Var}(Y) \approx \left(\frac{1}{f'(N)}\right)^2 \mathrm{Var}(Y)$$

bioRxiv preprint doi: https://doi.org/10.1101/2025.09.08.674884; this version posted May 21, 2026. The copyright holder for this preprint (which was not certified by peer review) is the author/funder, who has granted bioRxiv a license to display the preprint in perpetuity. It is made available under a CC-BY 4.0 International license.

$$\approx \frac{\sum_{j=1}^{K}\left[e^{-p_j N} - e^{-2p_j N}\right] - N\sum_{j\neq k}\left[p_j p_k e^{-N(p_j+p_k)}\right]}{\left(\sum_{j=1}^{K} p_j e^{-p_j N}\right)^2} \tag{25}$$

This enables us to compute confidence intervals for our predictions $\hat{N}(y)$, leveraging the asymptotic normality of Y [15] discussed in the next section.

S5.3 Runtime and memory analysis of Method-of-Moments Estimator

We analyze the runtime and memory complexity of Algorithms 1 and 2 in terms of the total number of UMIs K and the maximum observed count $Y_{\max}$. Let $\tilde{Y}_{\max} = \min(Y_{\max}, K-1)$.

Algorithm 2 (Precomputation).
The repeat loop (lines 3–6) iterates at most $n_{\max}$ times, where

$$n_{\max} \leq \left\lceil \frac{\log(1-\tilde{Y}_{\max}/K)}{\log(1-p_{\min})} \right\rceil, \tag{26}$$

and $p_{\min} = \min_j p_j$. Each iteration updates the running powers $(1-p_j)^n$ for $j = 1,\ldots,K$ via a single multiplication per coordinate j and accumulates Y_n, requiring $O(K)$ compute per iteration. The subsequent for loop (lines 9–13) sweeps over $y = 1,\ldots,\tilde{Y}_{\max}$ while advancing a pointer n_{ptr} that increases monotonically from 1 to at most $n_{\max}$. We have that $n \leq n_{\max}$, and so this loop runs at most $O(n_{\max})$ times, with each interpolation step costing $O(1)$. The overall time complexity of Algorithm 2 is therefore

$$O(K \cdot n_{\max}), \tag{27}$$

and it requires $O(K + n_{\max})$ memory to store the running powers $(1-p_j)^n$ and the sequence $Y_1,\ldots,Y_{n_{\max}}$. Note that this memory cost can be made independent of K by simply recomputing all terms at each iteration. In the uniform case $p_j = 1/K$, the bound simplifies to $n_{\max} = \lceil \log(1-\tilde{Y}_{\max}/K)/\log(1-1/K)\rceil$.

Algorithm 1 (Collision-aware estimator).
Given the precomputed lookup table $\hat{N}(0),\ldots,\hat{N}(Y_{\max})$, correcting each observed count requires a single $O(1)$ table lookup, so the per-entry cost of applying the correction is constant. The total end-to-end complexity is

$$O(K \cdot n_{\max} + \tilde{Y}_{\max} + nd), \tag{28}$$

where nd can be replaced with the sparsity S by simply applying this estimator to the nonzero entries. For e.g. $k = 6$ with $K = 4096$, and $\tilde{Y}_{\max} \approx 10^4$ for most datasets, the precomputation cost is modest relative to the size of the counts matrix.

S6 Asymptotic normality analysis

In this section, we leverage the asymptotic normality of Y to analyze the performance of our estimators in greater detail. For simplicity and concreteness, we focus on the uniform UMI setting, where $p_j = 1/K$ for all $j \in [K]$. All results extend naturally to the nonuniform case (and the nonuniform method-of-moments estimator), but with more cumbersome expressions and less clear insights.

It is known that Y is asymptotically normal whenever $\text{Var}(Y) \to \infty$ [15, 23]. Thus, for $N, K \to \infty$ we have under this condition that:

$$Y \sim \mathcal{N}\big(\mathbb{E}[Y], \text{Var}(Y)\big),$$

with variance (from Equation (4)), simplified for the uniform case to:

$$\begin{aligned}\text{Var}(Y) &= K\left(1-\frac{1}{K}\right)^N + K(K-1)\left(1-\frac{2}{K}\right)^N - K^2\left(1-\frac{1}{K}\right)^{2N} \\ &\approx e^{-2N/K}\left[K\left(e^{N/K}-1\right) - N\right]\end{aligned} \tag{29}$$

bioRxiv preprint doi: https://doi.org/10.1101/2025.09.08.674884; this version posted May 21, 2026. The copyright holder for this preprint (which was not certified by peer review) is the author/funder, who has granted bioRxiv a license to display the preprint in perpetuity. It is made available under a CC-BY 4.0 International license.

This variance vanishes for large N; specifically, as we show in Proposition 2, for $N \geq cK \log K$ with $c > 1$ we have $\mathrm{Var}(Y) \to 0$ as $N, K \to \infty$. In this case, Y converges to a point mass at K, and so asymptotic normality does not hold.

For N below this threshold, we define $f(N) = K\left(1 - e^{-N/K}\right) \approx \mathbb{E}[Y]$. This approximation uses $\log(1 - 1/K) \approx -1/K$ for $K \gg 1$, and is asymptotically tight. Then

$$f(N) = K\left(1 - e^{-N/K}\right), \qquad f'(N) = e^{-N/K}.$$

With this function-based reparameterization, we can express our (uniform UMI) estimator as $\hat{N}(Y) \approx f^{-1}(Y)$, up to the $\log(1 - 1/K)$ and exponential approximations. This enables us to approximate the bias and variance of our estimator using the delta method.

S6.1 Asymptotic variance of MoM estimator

Since $\hat{N}(Y) \approx f^{-1}(Y)$, by the delta method we have

$$\mathrm{Var}\left(\hat{N}(Y)\right) \approx \left(g'(f(N))\right)^2 \mathrm{Var}(Y), \qquad \text{where } g = f^{-1}.$$

Since

$$g'(f(N)) = \frac{1}{f'(N)} = e^{N/K},$$

we obtain the asymptotic variance

$$\begin{aligned}\mathrm{Var}\left(\hat{N}(Y)\right) &\approx \frac{1}{\left(f'(N)\right)^2}\mathrm{Var}(Y) \\ &\approx e^{2N/K}\mathrm{Var}(Y) &(30)\\ &= K\left(e^{N/K} - 1\right) - N. &(31)\end{aligned}$$

This expression governs the behavior of $\mathrm{Var}(\hat{N})$ across the relevant regimes of N:

$$\mathrm{Var}(\hat{N}) = \begin{cases} \Theta\left(\dfrac{N^2}{K}\right), & N = O(K), \\ \Theta\left(Ke^{N/K}\right), & N = \omega(K), N = o(K \log K), \\ o(1), & N \geq cK \log K \text{ for } c > 1. \end{cases}$$

In particular, for $N \leq cK \log K$ with any $c < 1$, the delta–method approximation is accurate and (31) describes the variance growth of the collision-aware estimator.

Most helpful for comparative analysis when $N = o(K \log K)$ is the characterization in Equation (30): $\mathrm{Var}\left(\hat{N}\right) \approx e^{2N/K}\mathrm{Var}(Y)$. This indicates that, firstly, the variance of our estimator is always larger than the variance of Y, the naive estimator. However, even for large N approaching K, the variance of our estimator is only a constant factor larger than the variance of Y. Concretely, for $N = K$, we have that $\mathrm{Var}\left(\hat{N}(Y)\right)$ is only a factor of $e^2 \approx 7.39$ larger than $\mathrm{Var}(Y)$. It is not until N approaches $K \log K$ that the variance of our estimator becomes significantly larger than the variance of Y, where in the regime of $N = cK \log K$ for $c < 1$, the variance of our estimator is approximately $K^{2c}\mathrm{Var}(Y)$. However, in this regime, Y is already highly concentrated around K, and so the variance of Y is decaying as $e^{-N/K}$. Next, we analyze the bias of our estimator, showing that this small increase in variance enables a significant reduction in bias compared to the naive estimator.

S6.2 Asymptotic bias of MoM estimator

We can similarly approximate the bias of our estimator $\hat{N}(Y)$ using a second-order delta method, working directly with the parameterization $f(N) = Y_N$, where $Y_N = \mathbb{E}[Y]$.

We compute the second derivative of the inverse function $g = f^{-1}$ using the chain rule:

$$g'(y) = \frac{1}{f'(g(y))}, \qquad g''(y) = -\frac{f''(g(y))}{\left(f'(g(y))\right)^3}.$$

bioRxiv preprint doi: https://doi.org/10.1101/2025.09.08.674884; this version posted May 21, 2026. The copyright holder for this preprint (which was not certified by peer review) is the author/funder, who has granted bioRxiv a license to display the preprint in perpetuity. It is made available under a CC-BY 4.0 International license.

Evaluating at $y = f(N)$ (so $g(f(N)) = N$) yields

$$g'(f(N)) = \frac{1}{f'(N)}, \qquad g''(f(N)) = -\frac{f''(N)}{\big(f'(N)\big)^3}.$$

The second-order delta method gives

$$\mathbb{E}[\hat{N}(Y)] \approx g\big(f(N)\big) + \frac{1}{2}g''\big(f(N)\big)\mathrm{Var}(Y) = N - \frac{1}{2}\frac{f''(N)}{\big(f'(N)\big)^3}\mathrm{Var}(Y),$$

so the bias of the method-of-moments estimator is

$$\mathrm{Bias}\big(\hat{N}(Y)\big) \triangleq \mathbb{E}[\hat{N}(Y)] - N \approx -\frac{1}{2}\frac{f''(N)}{\big(f'(N)\big)^3}\mathrm{Var}(Y). \tag{32}$$

Using the approximations above,

$$f'(N) = e^{-N/K}, \qquad f''(N) = -\frac{1}{K}e^{-N/K},$$

and so

$$\begin{aligned}\mathrm{Bias}\big(\hat{N}(Y)\big) &\approx -\frac{1}{2}\frac{-e^{-N/K}/K}{\big(e^{-N/K}\big)^3}\mathrm{Var}(Y)\\ &= \frac{1}{2}\frac{e^{2N/K}}{K}\mathrm{Var}(Y).\end{aligned}$$

Using the variance approximation from (29), for when $N < K\log K$,

$$\mathrm{Var}(Y) \approx Ke^{-2N/K}\big(e^{N/K} - 1 - N/K\big),$$

we obtain the explicit bias, noting that when $N \geq K\log K$, $Y = K$ with high probability (Proposition 2), and so the bias is $\Theta(N)$:

$$\begin{aligned}\mathrm{Bias}\big(\hat{N}(Y)\big) &\approx \frac{1}{2}\Big(e^{N/K} - 1 - N/K\Big) \qquad (33)\\ &= \begin{cases}\Theta\left(\frac{N^2}{K^2}\right), & \text{when } N = O(K),\\ \Theta\left(e^{N/K}\right), & \text{when } N = \Omega(K) \text{ and } N < K\log K,\\ \Theta\left(N\right), & \text{when } N \geq cK\log K \text{ for } c > 1.\end{cases}\end{aligned}$$

For $N \leq K$, the bias is smaller than both $\sqrt{\mathrm{Var}(\hat{N}(Y))} = \Theta\big(N/\sqrt{K}\big)$ and the $O(N^2/K)$ bias of the naive estimator. Thus, in all pre-saturation regimes, the MoM estimator is only very mildly upward biased. However, once $N > K$, the bias starts increasing exponentially with N/K, maxing out at $\Theta(N)$ once N reaches $K\log K$ and Y saturates.

S6.3 Extension to nonuniform UMIs

For a nonuniform UMI distribution $\boldsymbol{p}$, we can define f accordingly and proceed with the analysis:

$$\begin{aligned}f(N) &= K - \sum_{j=1}^{K}(1-p_j)^N \approx K - \sum_{j=1}^{K} e^{-p_j N},\\ f'(N) &= -\sum_{j=1}^{K}\log(1-p_j)(1-p_j)^{N-1} \approx \sum_{j=1}^{K} p_j e^{-p_j N},\end{aligned}$$

bioRxiv preprint doi: https://doi.org/10.1101/2025.09.08.674884; this version posted May 21, 2026. The copyright holder for this preprint (which was not certified by peer review) is the author/funder, who has granted bioRxiv a license to display the preprint in perpetuity. It is made available under a CC-BY 4.0 International license.

where the approximations use $\log(1+x) \approx x$ and $(1-x)^N \approx e^{-xN}$ for small x, and that N is large. Differentiating once more gives

$$f''(N) \approx -\sum_{j=1}^{K} p_j^2 e^{-p_j N}.$$

Let $g = f^{-1}$ denote the inverse function. Then $\hat{N}(Y) \approx g(Y)$, and Y is approximately Gaussian with mean $f(N)$ and variance $\mathrm{Var}(Y)$. Using the approximations above,

$$f'(N) \approx \sum_{j=1}^{K} p_j e^{-p_j N}, \qquad f''(N) \approx -\sum_{j=1}^{K} p_j^2 e^{-p_j N},$$

and (32) becomes

$$\mathrm{Bias}\big(\hat{N}(Y)\big) \approx \frac{1}{2} \frac{\sum_{j=1}^{K} p_j^2 e^{-p_j N}}{\left(\sum_{j=1}^{K} p_j e^{-p_j N}\right)^3} \mathrm{Var}(Y). \tag{34}$$

Similarly,

$$\mathrm{Var}\left(\hat{N}(Y)\right) \approx \frac{1}{\big(f'(N)\big)^2} \mathrm{Var}(Y) \tag{35}$$

$$\approx \frac{1}{\left(\sum_{j=1}^{K} p_j e^{-p_j N}\right)^2} \mathrm{Var}(Y). \tag{36}$$

S7 Optimality of method-of-moments estimator for uniform UMIs

On its surface, our proposed estimator seems quite simplistic. It only matches the first moment of N, and fails to take into account any higher order moments of Y. Additionally, since the estimator is a convex function of y, by Jensen's inequality $\mathbb{E}[\hat{N}(Y)] \geq \hat{N}(\mathbb{E}[Y])$, implying that $\hat{N}(Y)$ will overestimate N. However, as we show, this estimator yields good estimation up until the threshold of impossibility.

S7.1 MSE analysis and comparison

One interesting note is the directions of the biases. The naive estimator is always negatively biased, as collisions cause undercounting. On the other hand, our estimator is positively biased, as it is a convex function of Y, where our moment matching condition combined with Jensen's inequality imply that $\mathbb{E}[\hat{N}(Y)] \geq \hat{N}(\mathbb{E}[Y]) = N$.

Recall that the variance of Y is

$$\mathrm{Var}\,(Y) \approx e^{-2N/K}\left[K\left(e^{N/K}-1\right)-N\right] \tag{37}$$

$$= \begin{cases} \Theta\left(\frac{N^2}{K}\right), & \text{when } N = O(K), \\ \Theta\left(Ke^{-N/K}\right), & \text{when } N = \Omega(K). \end{cases}$$

The bias of the naive estimator is:

$$|\mathbb{E}[Y] - N| \approx \left|K\left(1-e^{-N/K}\right)-N\right| \tag{38}$$

$$= \begin{cases} \Theta\left(\frac{N^2}{K}\right), & \text{when } N = O(K), \\ \Theta\left(N\right), & \text{when } N = \Omega(K). \end{cases}$$

The MSE of the naive estimator is always dominated by the squared bias term. Splitting our analysis into regimes (tabulated in Table 1), we have that:

bioRxiv preprint doi: https://doi.org/10.1101/2025.09.08.674884; this version posted May 21, 2026. The copyright holder for this preprint (which was not certified by peer review) is the author/funder, who has granted bioRxiv a license to display the preprint in perpetuity. It is made available under a CC-BY 4.0 International license.

1. $N = o(\sqrt{K})$. Here, both estimators have vanishing bias and variance.
2. $N = o(K), N = \Omega(\sqrt{K})$. Here, the $\Theta(N^2/K)$ bias of the naive estimator starts growing, leading to an MSE dominated by the squared bias of order $\Theta(N^4/K^2)$. Our MoM estimator still has vanishing bias, but $\mathrm{Var}(Y) \approx O(N^2/K)$. This retains MSE sublinear in N.
3. $N = \Omega(K), N = o(K \log K)$. The naive estimator already has linear bias, and so an MSE of order N^2. Here, $\mathrm{Var}(Y) \approx Ke^{-N/K}$ is decaying, but our estimator now has inflated variance, leading to $\mathrm{Var}\left(\hat{N}(Y)\right) = \Theta(Ke^{N/K})$, dominating the MSE of our MoM estimator.
4. $N \geq cK \log K$ for $c > 1$. Here, Y has saturated (by Proposition 2), and so both estimators have bias and MSE on the order of N^2.

Regime \ Estimator	Naive Estimator			Collision-aware Estimator		
	Bias	Variance	MSE	Bias	Variance	MSE
$N = o(\sqrt{K})$	$o(1)$	$o(1)$	$o(1)$	$o(1)$	$o(1)$	$o(1)$
$N = o(K), N = \Omega(\sqrt{K})$	$\Theta\left(N^2/K\right)$	$\Theta\left(N^2/K\right)$	$\Theta\left(N^4/K^2\right)$	$o(1)$	$\Theta\left(N^2/K\right)$	$\Theta\left(N^2/K\right)$
$N = o(K \log K), N = \Omega(K)$	$\Theta(N)$	$\Theta\left(Ke^{-N/K}\right)$	$\Theta(N^2)$	$\Theta(e^{N/K})$	$\Theta(Ke^{N/K})$	$\Theta(Ke^{N/K})$
$N \geq cK \log K$ for $c > 1$	$\Theta(N)$	o(1)	$\Theta(N^2)$	$\Theta(N)$	o(1)	$\Theta(N^2)$

Table S1: Comparison of Bias, Variance, and MSE for naive and collision-aware estimators across different regimes of N. Table replicated from Table 1 in the main text for convenience.

This highlights that when $N = O(\sqrt{K})$ both estimators attain vanishing MSE. However, for larger N, the naive estimator has increasing bias which dominates the MSE. When N falls between K and $K \log K$, the MSE of the naive estimator already scales as N^2, dominated by the bias, while our estimator performs slightly worse than the earlier MSE of $\Theta(N)$, but still sub-quadratic. The high level takeaway is that in order to minimize the MSE, K is currently taken so that $N \leq K^{2/3}$ to avoid the large bias of the naive estimator. However, K can in fact be selected such that N is a constant multiple of K, in which case the MSE is still $O(N)$. Next, we discuss impossibility results and lower bounds in this setting.

S7.2 Impossibility beyond $N > K \log K$: proof of Proposition 2

From the classical coupon collector problem, it is known that the expected number of balls required until all bins are filled is $N = K \log K + O(K)$. This threshold is tight: taking N to be larger than $K \log K$ by any multiplicative constant yields vanishing (with K) probability of observing $Y < K$.

Proposition 2. *For $N = cK \log K$ with $c > 1$, $\mathbb{P}(Y = K) \geq 1 - K^{1-c}$.*

Proof of Proposition 2 Define the indicator random variables $Z_j = \mathbb{1}\{\cup_i \{X_i = j\}\}$, whether bin j is filled, for $j \in [K]$. Then, $Y = \sum_j Z_j$.

$$\begin{aligned}\mathbb{P}(Y < K) &= \mathbb{P}\left(\cup_j \{Z_j = 0\}\right) \\ &\leq K\mathbb{P}\left(\{Z_1 = 0\}\right) \\ &= K\left(1 - 1/K\right)^N \\ &\leq Ke^{-N/K} \\ &= K^{1-c} \end{aligned} \tag{39}$$

where a union bound is used, followed by the inequality that $1 - x \leq e^{-x}$. □

Since Y will be equal to K with high probability, we cannot distinguish between $N = 2K \log K$ and $N' = K^3$, for example, and so N cannot be estimated to any nontrivial accuracy. Extending this to the nonuniform p setting is difficult, without a closed form solution. Defining $p_{\min} = \min_j p_j$, we see that $N = \Omega(1/p_{\min})$ is necessary, as otherwise the UMI corresponding to $p_{\min}$ will not have been observed with high probability. Conversely, $N = O(\log(K)/p_{\min})$ is sufficient, by a similar union bounding argument. Again, by concavity, $N = \Omega(K \log K)$ is necessary.

bioRxiv preprint doi: https://doi.org/10.1101/2025.09.08.674884; this version posted May 21, 2026. The copyright holder for this preprint (which was not certified by peer review) is the author/funder, who has granted bioRxiv a license to display the preprint in perpetuity. It is made available under a CC-BY 4.0 International license.

S7.2.1 Extending saturation threshold beyond $K \log K$ by adjusting the UMI distribution

The above observations regarding the saturation threshold scaling as $1/p_{\min}$ imply that we can increase our threshold for feasible estimation by decreasing the minimum probability. A natural question is then: how far can we extend our nontrivial estimation? As we drop $p_{\min} \to 0$, Y will not saturate until $1/p_{\min}$, but gain increased variance before. Theoretically, given a known distribution of true UMI counts (N), or a range of feasible N, one could compute the best UMI distribution with respect to this Bayes Risk or Minimax Risk. Even by taking a simple setting, where per-nucleotide probabilities are $[1-x, 1-x, 1-x, 3x]$ for [A,C,G,T], with $x \in [0, 1/3]$, by taking x to 0 we can guarantee that Y will not saturate, and that via the asymptotic normality argument below, we can compute the MSE for any fixed x and N.

S7.3 Cramér–Rao lower bound in binomial setting

To begin, N is a discrete parameter, and so we technically cannot directly apply the Cramér–Rao lower bound. However, since the likelihood is a continuous function of N, we can instead perform inference when $N \in \mathbb{R}_+$ and then apply the Cramér–Rao lower bound to the continuous function.

Recall the indicator random variable based definition of Y, with $Z_j = \mathbb{1}\{\cup_i \{X_i = j\}\}$, whether bin j is filled, for $j \in [K]$, and $Y = \sum_j Z_j$. Here,

$$p(N) = \mathbb{P}(Z_1 = 1) = 1 - \left(1 - \frac{1}{K}\right)^N.$$

In our setting these Z_j are correlated, making the analysis difficult, so we approximate Y by $\tilde{Y}$, a binomial where the Z_j are independent:

$$\mathbb{P}(\tilde{Y} = y; N) = \binom{K}{y} p(N)^y \Big(1 - p(N)\Big)^{K-y}.$$

The mean of $\tilde{Y}$ matches Y, as:

$$\mathbb{E}[\tilde{Y}] = Kp(N) = K\left[1 - \left(1 - \frac{1}{K}\right)^N\right],$$

and its variance is (in this binomial approximation)

$$\sigma_N^2 = \mathrm{Var}(\tilde{Y}) = Kp(N)\Big[1 - p(N)\Big],$$

which is approximately the variance of Y.

The log-likelihood for $\tilde{Y}$ is then

$$\ell(N; y) = \ln \binom{K}{y} + y \ln p(N) + (K - y) \ln\Big(1 - p(N)\Big).$$

The Fisher information is defined as

$$I(N) = \mathbb{E}\left[\left(\frac{\partial \ell}{\partial N}\right)^2\right] = \mathbb{E}\left[\left(\frac{\tilde{Y} - Kp(N)}{p(N)(1 - p(N))} \frac{dp}{dN}\right)^2\right].$$

Plugging in for the variance of $\tilde{Y}$ in the numerator, it follows that

$$I(N) = \frac{\mathrm{Var}(\tilde{Y})}{[p(N)(1 - p(N)]^2} \left(\frac{dp}{dN}\right)^2 = \frac{K}{p(N)(1 - p(N))} \left(\frac{dp}{dN}\right)^2.$$

bioRxiv preprint doi: https://doi.org/10.1101/2025.09.08.674884; this version posted May 21, 2026. The copyright holder for this preprint (which was not certified by peer review) is the author/funder, who has granted bioRxiv a license to display the preprint in perpetuity. It is made available under a CC-BY 4.0 International license.

For large K, we may use the approximations

$$\left(1 - \frac{1}{K}\right)^N \approx e^{-N/K}, \quad \ln\left(1 - \frac{1}{K}\right) \approx -\frac{1}{K},$$

so that

$$p(N) \approx 1 - e^{-N/K}, \qquad \frac{dp}{dN} \approx e^{-N/K} \ln\left(1 - \frac{1}{K}\right) \approx -\frac{e^{-N/K}}{K}.$$

Then, the Fisher information becomes

$$I(N) \approx \frac{K}{\left(1 - e^{-N/K}\right)e^{-N/K}} \left(\frac{1}{Ke^{N/K}}\right)^2 = \frac{1}{K\left(e^{N/K} - 1\right)}.$$

This allows us to state our Cramér–Rao lower bound:

Theorem 1. *Any unbiased estimator $\tilde{N}$ of N, given observation $\tilde{Y}$, satisfies*

$$\mathrm{Var}(\tilde{N}) \geq \frac{1}{I(N)} \approx K\left(e^{N/K} - 1\right).$$

Our method-of-moments estimator matches this lower bound in this simplified setting (Equation (31)), highlighting the near-optimality of our estimator. Observe that our estimator has nontrivial bias for $N = \Omega(K)$, and so this lower bound does not directly apply in this regime, but for $N = o(K)$ our estimator is asymptotically unbiased. Evaluating this expression, we have that as N approaches $K \ln K$, we have $e^{N/K} \approx K$, so that $\mathrm{Var}(\hat{N}) = \Omega(K^2)$, indicating a dramatic increase in estimation error. In this regime, where nearly all bins are occupied, even small differences in Y lead to large differences in $\hat{N}(Y)$, and no estimator can achieve significantly lower variance than the approximate lower bound. Our method-of-moments estimator achieves this approximate lower bound up to a constant factor, and so is near-optimal in this regime.
